# A Genome-Wide RNAi Screen Identifies Regulators of Cholesterol-Modified Hedgehog Secretion in *Drosophila*

**DOI:** 10.1371/journal.pone.0033665

**Published:** 2012-03-14

**Authors:** Reid Aikin, Alexandra Cervantes, Gisela D'Angelo, Laurent Ruel, Sandra Lacas-Gervais, Sébastien Schaub, Pascal Thérond

**Affiliations:** 1 CNRS UMR 7277, Inserm UMR 1091, Institut de Biologie Valrose (iBV), Centre de Biochimie, Nice, France; 2 Université de Nice-Sophia Antipolis, Nice, France; 3 Centre Commun de Microscopie Appliquée (CCMA), Université de Nice-Sophia Antipolis, Nice, France; University of Dayton, United States of America

## Abstract

Hedgehog (Hh) proteins are secreted molecules that function as organizers in animal development. In addition to being palmitoylated, Hh is the only metazoan protein known to possess a covalently-linked cholesterol moiety. The absence of either modification severely disrupts the organization of numerous tissues during development. It is currently not known how lipid-modified Hh is secreted and released from producing cells. We have performed a genome-wide RNAi screen in *Drosophila melanogaster* cells to identify regulators of Hh secretion. We found that cholesterol-modified Hh secretion is strongly dependent on coat protein complex I (COPI) but not COPII vesicles, suggesting that cholesterol modification alters the movement of Hh through the early secretory pathway. We provide evidence that both proteolysis and cholesterol modification are necessary for the efficient trafficking of Hh through the ER and Golgi. Finally, we identified several putative regulators of protein secretion and demonstrate a role for some of these genes in Hh and Wingless (Wg) morphogen secretion *in vivo*. These data open new perspectives for studying how morphogen secretion is regulated, as well as provide insight into regulation of lipid-modified protein secretion.

## Introduction

Secreted morphogens play a critical role in the organization of tissues in a developing organism and in the regulation of tissue homeostasis in adults. While disrupted morphogen signalling is associated with various birth defects, many types of cancer involve aberrant morphogen signalling [1]. Morphogens are able to form concentration gradients originating from their source of production, providing positional information to receiving cells based on their distance from the morphogen source. Morphogens posses the ability to spread over large distances - up to 300 µm in the case of Hh in the vertebrate ventral neural tube and 12–15 cell diameters in *Drosophila* imaginal discs [2]. Consequently, the spatial and temporal regulation of morphogen levels and spreading in a cellular field must be tightly regulated.

Several morphogens have hydrophobic post-translational modifications that are essential for the spatial control of the morphogen signal. In other contexts, covalent lipid modifications can regulate the subcellular trafficking of proteins. For example, the attachment of a glycosylphosphatidylinositol (GPI)-anchor is required for efficient ER-to-Golgi trafficking of certain proteins and results in their sorting into distinct vesicles upon exit from the ER [3]. Similarly, palmitoylation affects the subcellular trafficking of a number of proteins [4]. As hydrophobic covalent modifications appear to confer increased membrane association, it is intriguing that some secreted morphogens such as the long-range activators Wg, Spitz, and Hh contain hydrophobic post-translational modifications essential for their spatial distribution. The mechanisms by which lipid modifications affect morphogen subcellular trafficking and release are not clear.

Hh is synthesized as a precursor form composed of an N-terminal signalling domain and a C-terminal catalytic domain. In an autoproteolytic reaction mediated by the C-terminal domain, the covalent addition of a cholesterol molecule to the N-terminal domain results in the cleavage and release of the C-terminal domain [5], [6]. The N-terminal domain is also modified by a palmitate [7], resulting in a highly hydrophobic protein able to form multimers [8], [9]. It is not known in which subcellular compartment(s) these lipid modifications occur. There is evidence that the cholesterol moiety affects the intracellular trafficking of Hh, as a mutant form of Hh lacking the cholesterol adduct shows altered subcellular distribution [10]. In addition, the C-terminal domain regulates the subcellular trafficking of Hh in photoreceptor neurons [11]. Various genetic screens in *Drosophila* have identified only one gene required to release cholesterol-modified Hh from producing cells: the multipass transmembrane protein Dispatched (Disp) [12]. However, the role of Disp is unclear as there is still juxtacrine Hh signalling in the absence of Disp, suggesting that Hh is still secreted to the plasma membrane [13], [14]. Overall, very little is known about 1) where within producing cells Hh processing occurs, 2) the intracellular route taken by Hh, or 3) the cellular machinery required to secrete such a lipid-modified protein.

To gain insight into the cellular machinery required to secrete lipid-modified Hh protein, we performed a genome-wide RNAi screen in *Drosophila* S2 cells. We found that secretion of lipid-modified Hh is strongly dependent on COPI–mediated transport but not COPII, a feature that is dependent on the cholesterol modification of Hh. We also show that two variants of Hh, an uncleavable form and a non-cholesterol modified form, are less efficiently transported through the secretory machinery suggesting that proteolysis and cholesterol modification both play a role in the trafficking of Hh through the ER and Golgi. Finally, we identified several novel genes which affect Golgi morphology and general secretion. The overexpression of dsRNAs against some of these genes generated a substantial reduction of the non-autonomous activity of both Hh and Wg.

## Results

### Genome-wide RNAi screen for regulators of Hh secretion and release

**Figure 1:**
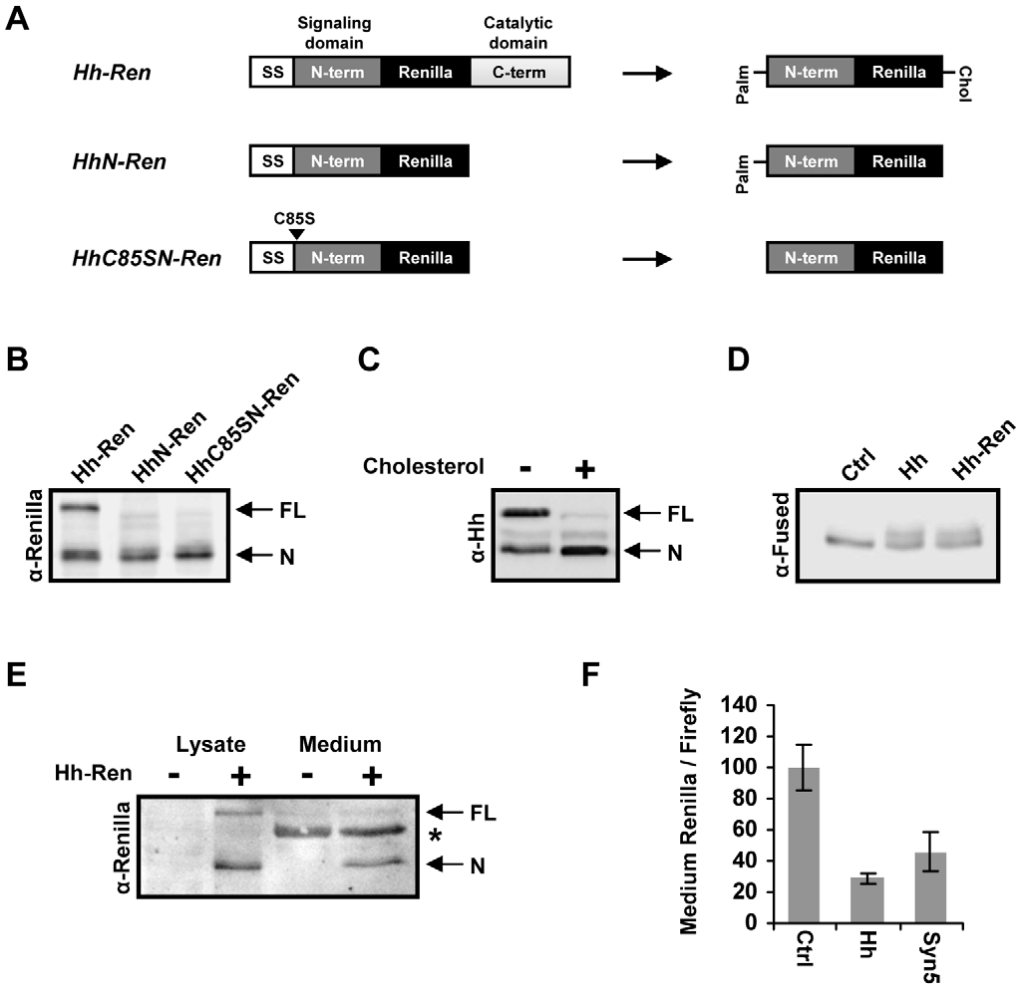
Generation of a secreted, biologically active Hh-*Renilla* fusion protein. (**A**) Schematic representation of the secreted *Hh-Ren* constructs. The coding sequence for *Renilla luciferase* was inserted upstream of the autoproteolytic cleavage site of Hh such that the processed N-terminal protein would be fused to *Renilla*. Hh-Ren fusions lacking either the cholesterol (*HhN-Ren*) or both cholesterol and palmitate modifications (*HhC85SN-Ren*) were also generated. (**B**) Western blot of cell lysates from S2 cells expressing different *Renilla* fusions constructs showing the full-length (FL) and N-terminal proteins (N). (**C**) Processing of Hh-Ren is increased by addition of cholesterol. S2 cells expressing Hh-Ren were cultured in the presence or absence of a cholesterol lipid concentrate. After 5 days, the cells were lysed and examined by western blot analysis using a Hh-specific antibody. (**D**) Hh-Ren activates the Hh signalling pathway. Lysates from cells expressing either wild-type Hh or Hh-Ren examined by western blot analysis for Fused (Fu) show a change in electromobility of Fu, indicative of Fu phosphorylation and pathway activation (Ctrl: untransfected S2 cells). (**E**) Only processed Hh-Ren is secreted. Normal S2 cells or cells expressing Hh-Ren were cultured in serum-free medium to allow detection of Hh-Ren in conditioned medium (which is normally masked by serum on a WB). After 24 h, medium was collected and cells were lysed and examined by western blot analysis using anti-*Renilla*. The asterisks (*) indicates a non-specific band in the conditioned medium. (**F**) Effect of RNAi on a stable cell line co-expressing Hh-Ren and a cytoplasmic firefly *luciferase*. Cells were treated with dsRNA against Ci (Ctrl), which is not expressed in S2 cells, Hh or syntaxin 5 (Syn5) for 5 days, at which point the medium was changed and cells were cultured for an additional 24 h. The bars represent the mean medium *Renilla* activity normalized by the lysate firefly activity ± SD.

To identify genes required for secretion and release of Hh we performed an RNAi screen in *Drosophila* cells. S2 cells transfected with Hh construct secrete biologically active [15], multimeric [8], [9], lipid-modified Hh [5], [7], indicating that these cells posses the cellular machinery required to process and release lipid-modified Hh. In addition, S2 cells can produce Hh multimers with similar size characteristics to those found in *Drosophila* embryos [8], [9]. To allow quantification of Hh secretion, the coding sequence for *Renilla luciferase* (Ren) was inserted into the N-terminal signalling domain of Hh (Figure 1A), at the same location where insertion of a GFP moiety or an HA tag resulted in a functional protein *in vivo* [12], [16]. Upon Hh autocatalytic processing, the full length Hh-Ren protein (∼80 kDa) undergoes proteolysis to yield the N-terminal Hh signalling domain fused to *Renilla* (∼56 kDa) (Figure 1B). Addition of cholesterol to the culture medium increased the processing of Hh-Ren, indicating that this construct is cholesterol modified (Figure 1C). Moreover, only the processed 56 kDa N-terminal form of Hh-Ren could be detected in conditioned medium of Hh-Ren expressing cells (Figure 1E). Hh-Ren was able to induce, to the same degree as Hh, an electromobility shift of Fused kinase, indicative of pathway activation [15], demonstrating that the Hh-Ren construct encodes a biologically active protein (Figure 1D). In order to perform a large scale RNAi screen, we generated an S2 cell line stably expressing Hh-Ren and a cytoplasmic firefly *luciferase* both under the control of the *actin* promoter. The firefly *luciferase* activity in the cell lysate served to normalize for differences in cell number and to identify dsRNAs that target key cellular processes affecting protein production. Treatment of these cells with dsRNA against Hh decreased *Renilla* activity in both the medium and cell lysates, and treatment with dsRNA against Syntaxin 5 (Syn5), a t-SNARE required for ER-Golgi transport, decreased the secretion of Hh-Ren without significantly affecting cytoplasmic firefly levels (Figure 1F).

**Figure 2:**
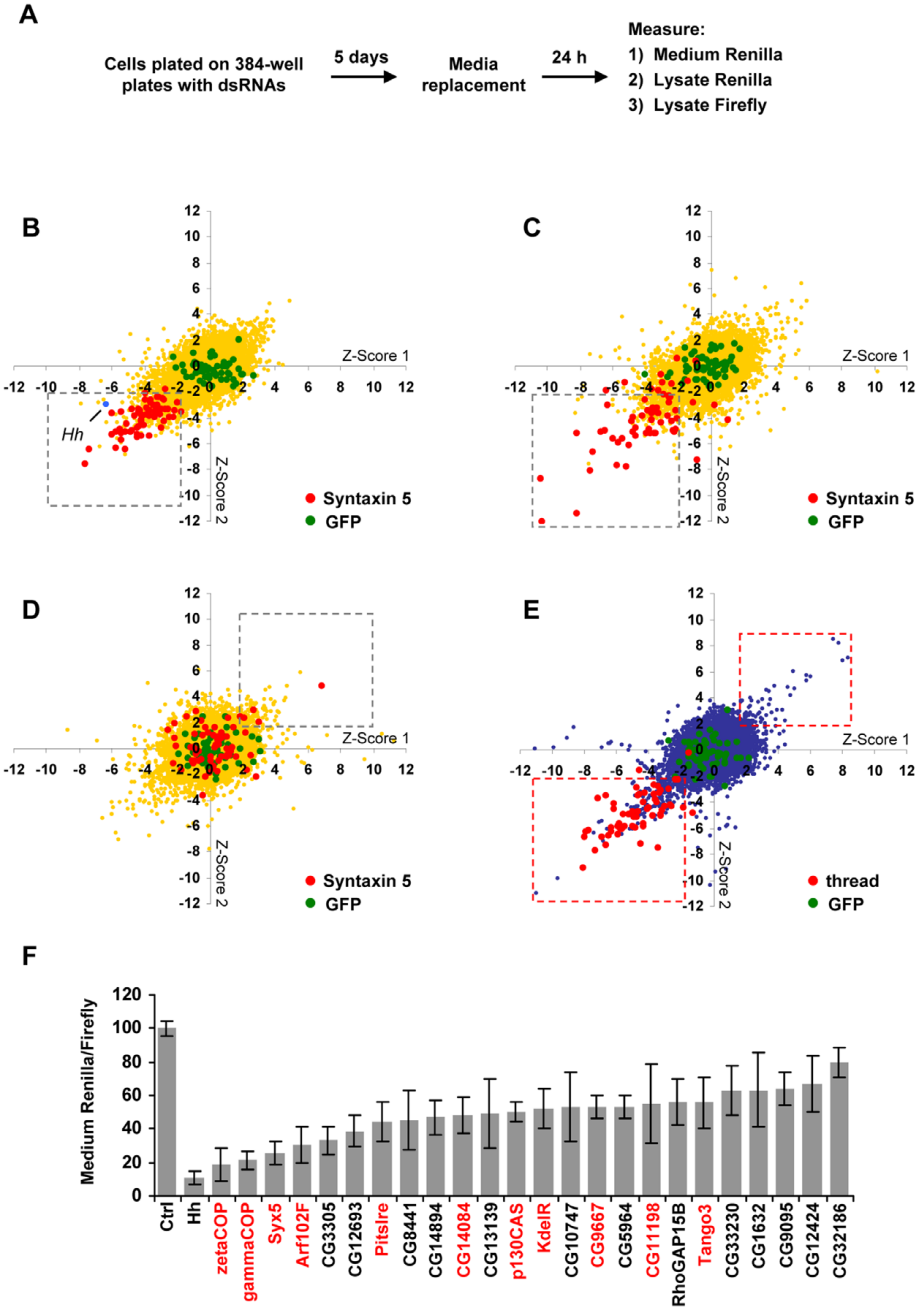
A genome-wide RNAi screen for regulators of Hh secretion and release. (**A**) RNAi screening procedure. Cells stably expressing Hh-Ren and a cytoplasmic firefly *luciferase* were treated with dsRNA for 5 days, at which point the culture medium was replaced. After 24 h, we measured the *Renilla* activity in the culture medium and both the *Renilla* and firefly activities in the cell lysates. (**B–D**) Scatter plots representing the duplicate z-scores for (B) medium *Renilla*/firefly (C) medium *Renilla*/lysate *Renilla*, and (D) lysate *Renilla*/firefly as the x and y coordinates for each dsRNA screened. The positive (Syn5; in red) and negative (GFP; in green) controls from each of the 58 plates are shown. Hits considered for further screening are indicated by the grey square. (**E**) Scatter plot representing the duplicate z-scores the firefly reading as the x and y coordinates for each dsRNA screened. The positive (thread; in red) and negative (GFP; in green) controls from each of the 58 plates are shown. Hits eliminated from further screening are indicated by the red squares. (**F**) Candidate regulators of Hh secretion confirmed by secondary screening. To confirm the effects of the candidate genes, new dsRNAs were synthesized which did not overlap with those used in the primary screen. S2 cells transiently transfected with an inducible pMT-Hh-Ren construct and a cytoplasmic firefly *luciferase* were cultured for 5 days with the indicated dsRNAs, at which point the medium was replaced with Cu-containing medium, and the cells were cultured for an additional 36 h. The bars represent the mean medium *Renilla* activity normalized by the lysate firefly activity ± SD. Genes identified in previous screens for regulators of general secretion in *Drosophila* cells [18], [55] are indicated in red. Ctrl: dsRNA against Ci.

Using this Hh-Ren and cytoplasmic firefly expressing cell line, we screened a library of ∼21,000 dsRNAs targeting over 95% of the annotated *Drosophila* genome in 384-well plate format (Figure 2A). Each plate contained dsRNAs against GFP and Syn5 as negative and positive controls respectively, and the entire screen was performed in duplicate. Three measures were taken for each well: *Renilla* activity of the media, *Renilla* activity of the lysate, and firefly activity of the lysate. We reasoned that dsRNAs targeting regulators of Hh-Ren secretion could affect one or more of the following ratios: A) *Renilla* activity in the medium normalized by firefly activity in the lysate, B) *Renilla* activity in the lysate normalized by firefly activity in the lysate, or C) *Renilla* activity in the medium divided by *Renilla* activity in the lysate (referred to from here on as ratio A, B, and C, respectively). The normalized values were transformed to z-scores, which indicate the number of standard deviations (SD) from the plate average. Based on the distribution of the Syn5 controls on each plate (Figure 2B–2D), we selected dsRNAs that caused an increase or decrease of greater than 2 SD from the plate average for both of the two replicate experiments in at least one of the above normalized ratios. While the Syn5 control scored more consistently as affecting the A and C ratios, we reasoned that other genes, acting at different points along the secretory pathway than Syn5, may score more strongly in ratio B depending on the ability of Hh-Ren to accumulate when blocked in various cellular compartments.

We eliminated dsRNAs that caused a decrease or increase in intracellular firefly of greater than 2 SD based on the distribution of the thread control (Figure 2E), the *Drosophila* inhibitor of apoptosis (dIAP), since normalization by such abnormal firefly values often resulted in artificially high or low normalized scores (Table S3). In addition, dsRNAs that significantly affect firefly activity may have an indirect effect on protein secretion by targeting genes involved in key cellular processes. Concordantly, our control thread dsRNA was often identified as a hit based on its affects on ratio A, B, or C, but would be eliminated since it reduced firefly activity by greater than 2 SD due to its negative effect on survival. While some known regulators of general secretion decreased firefly activity (beta'COP, sec61alpha), we reasoned that more specific regulators of Hh secretion would not likely have an effect on overall cell viability. We also noted that many dsRNAs with possible off-targets effects (OTE), where a minimum of 19 bp of the original amplicon sequence overlaps with a gene other than the intended target [17], often significantly decreased firefly activity. Indeed, 75% of the hits eliminated for their effect on cytoplasmic firefly had at least one 19 bp OT, which may increase their likelihood of affecting genes required for basic cell functions, thus leading to decreased firefly activity.

Following additional filtering (see Materials and Methods) we obtained 125 genes for secondary screening (Table S1). We performed secondary screening using S2 cells transiently transfected with an inducible pMT-Hh-Ren construct and a cytoplasmic firefly. By using an inducible promoter to drive the expression of Hh-Ren, we were able to allow RNAi-mediated target gene depletion prior to the induction of Hh-Ren expression. For these studies, we used dsRNAs that did not overlap with those of the primary screen. We identified with high confidence 24 genes whose depletion significantly affected Hh-Ren secretion (Figure 2F), 11 of which were identified in a previous RNAi-screen for regulators of general protein secretion [18], while the other genes have no previously described role in secretion. Although Disp mRNA is expressed in S2 cells (data not shown), no effect of Disp dsRNA was observed in our system (several independent dsRNAs were tested), possibly because Disp may only be required for trafficking of Hh in polarized cells and not for its secretion per se, as altered Hh subcellular distribution can be observed in Hh-producing cells of *disp* mutant embryos [10], and juxtacrine Hh signalling is still observed in the absence of Disp [13], [14]. Alternatively, high stability of Disp protein might preclude the dsRNA effect, a possibility that can only be addressed when an antibody recognizing the endogenous Disp is available. While components of the coat protein complex I (COPI) vesicle coat scored strongly, surprisingly no COPII components were identified. We therefore set out to 1) examine the role of COPI/COPII on Hh secretion, and 2) to characterize the role of novel genes identified in the screen on morphogen secretion *in vitro* and *in vivo*.

### Role of processing on Hh subcellular trafficking

**Figure 3:**
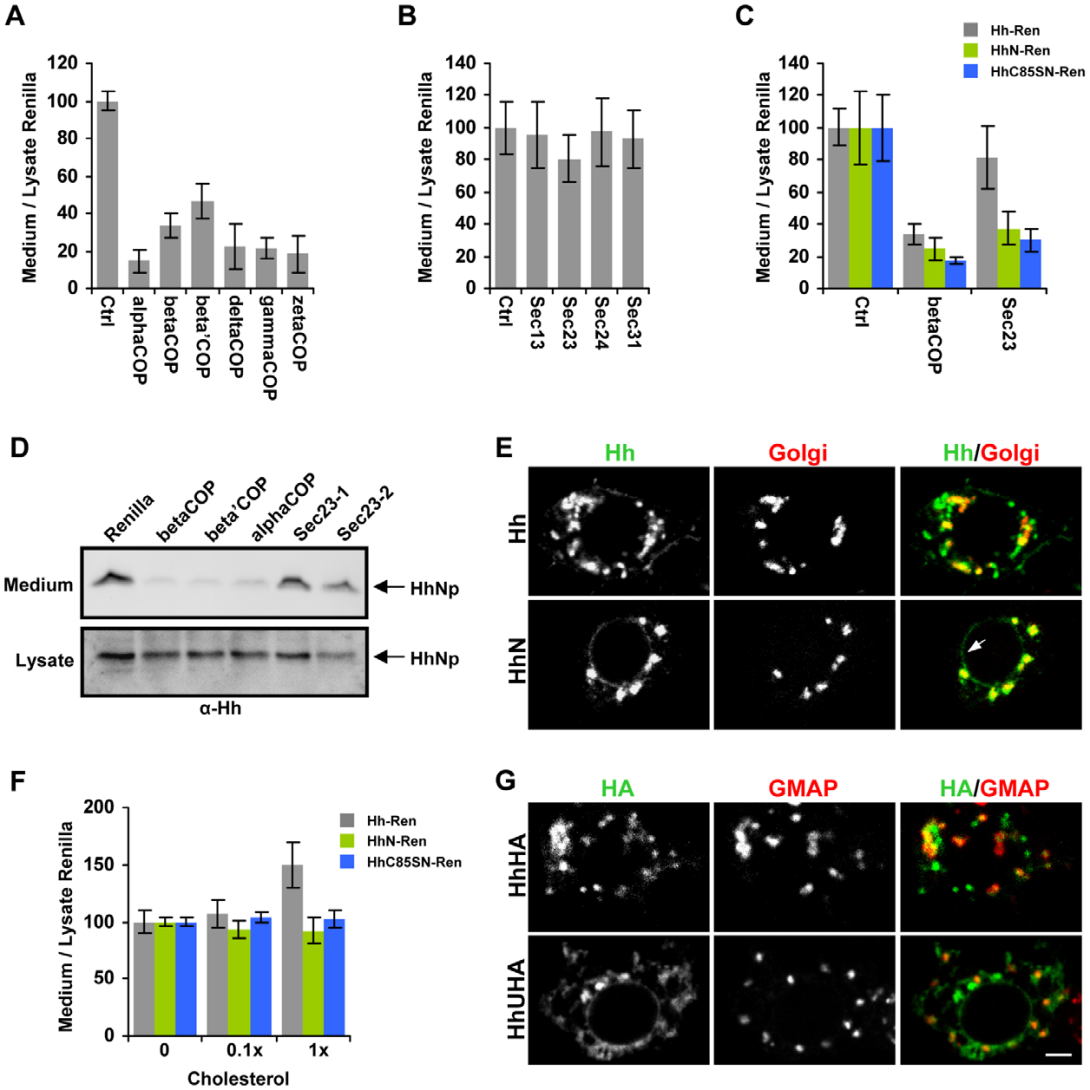
Cleavage and cholesterol modification affect the subcellular trafficking of Hh. (**A–C**) S2 cells were transiently transfected with pMT-Hh-Ren (**A,B**), pMT-HhN-Ren, or pMT-HhC85SN-Ren (**C**) and cultured for 5 days with the indicated dsRNAs and analyzed as in Figure 2F. The bars represent the mean medium/lysate *Renilla* activity ± SD. Similar results were obtained using several independent dsRNAs (Table S4). Ctrl: dsRNA against Ci. (**D**) COPI, but not COPII, is required for Hh secretion. S2R+ cells transiently transfected with an inducible pMT-Hh construct were treated with the indicated dsRNAs and cultured for 5 days, at which point the medium was replaced with Cu-containing medium, and the cells were cultured for an additional 36 h. Medium was collected and submitted to high speed spin and cells were lysed and examined by western blot analysis using anti-Hh. Similar results were obtained with two independent amplicons targeting Sec23: Sec23-1 (DRSC31248) and Sec23-2 (DRSC12387). (**E**) Lack of cholesterol modification causes Hh accumulation in the ER and Golgi. S2 cells were transfected with Hh, fixed and immunostained with anti-Hh (green) and anti-Golgi (red). In contrast to Hh, HhN was strongly localized to the Golgi and to the perinuclear ER (arrow). (**F**) Cholesterol promotes Hh secretion. S2 cells transiently transfected with an inducible pMT-Hh-Ren construct, pMT-HhN-Ren, or pMT-HhC85SN-Ren were cultured with the indicated amount of cholesterol concentrate and treated as in Figure 2F. The bars represent the mean medium/lysate *Renilla* activity ± SD. (**G**) Full length uncleaved Hh is retained in the ER. S2R+ cells were transfected with HhHA or HhUHA, fixed and immunostained with anti-HA (green) and anti-GMAP (red). HhUHA was retained in the ER, with very little co-localization with GMAP. Scale bars, 3 µm.

COPI and COPII coated vesicles play a critical role in traffic between the ER and Golgi [19]. Since several COPI components were identified as strong regulators of Hh-Ren secretion, we examined in greater detail the role of COP-mediated trafficking in Hh-Ren secretion. Upon retesting with several dsRNAs per gene (Table S4), we confirmed that the release of lipid-modified Hh appears strongly dependent on COPI, but not COPII, mediated transport (Figure 3A and 3B). We also examined the effect of COPI and COPII depletion on untagged Hh and found that dsRNAs targeting several COPI components severely reduced Hh levels in the medium, while two independent dsRNAs targeting the COPII component Sec23 did not significantly affect Hh levels in the medium (Figure 3D). While most secreted cargo exits the ER in COPII vesicles, there are examples of COPII-independent exit from the ER [20]. As there is evidence that some membrane-anchored proteins can be sorted upon exit from the ER [21], it is plausible that lipid-modification targets Hh to a COPII-independent route out of the ER.

We therefore examined whether the lipid modification of Hh affects the sensitivity to depletion of betaCOP (COPI) or Sec23 (COPII), vesicle component known to be susceptible to dsRNA treatment in S2 cells [18], and which were required for the release of a secreted form of *Renilla* (Figure S1A and S1B). In contrast to Hh-Ren, the secretion of forms of Hh lacking either the cholesterol moiety (HhN-Ren; see Figure 1A and 1B for construct details) or both cholesterol and palmitate modifications (HhC85SN-Ren) were susceptible to Sec23 dsRNA (Figure 3C). This finding demonstrates that Sec23 dsRNA is sufficient to block the release of non-cholesterol-modified proteins, confirming the efficiency of Sec23 RNAi in our system. A similar trend was also observed upon depletion of Sar1, a small GTPase required for the assembly of COPII vesicles, where Sar1 depletion inhibited the release of HhN-Ren to a greater extent than Hh-Ren (Figure S1C). However, unlike depletion of COPII components, Sar1 depletion did affect the release of Hh-Ren, possibly due to a role of Sar1 in ER homeostasis independent of its role in COPII vesicle formation [22]. These findings indicate that cholesterol-modification renders Hh less dependent on COPII-mediated transport and thus may alter Hh subcellular trafficking.

Using immunofluorescent microscopy, we examined the effect of cholesterol modification on the subcellular distribution of Hh. When expressed in S2 cells, wild-type Hh is found in the Golgi and also in numerous non-Golgi structures (Figure 3E). Similar results were observed with an HA-tagged version of Hh (top panels for Figure 3G). In contrast, HhN accumulated strongly in the Golgi as well as the perinuclear region (Figure 3E, bottom panels), indicative of ER retention in S2 cells [18], [23]. A similar difference in subcellular localization was also observed when comparing Hh-Ren and HhN-Ren or HhGFP and HhNGFP (Figure S2). Therefore, the lack of cholesterol-modification causes HhN to accumulate in the Golgi and ER.

These findings suggest that Hh processing might be required for efficient exit from the ER and Golgi. We therefore examined the subcellular localization of an uncleavable form of Hh (HhUHA) [11], which contains the C-terminal domain but cannot be cholesterol modified. Interestingly, HhUHA displayed a diffuse reticular staining pattern, often perinuclear, indicating that unprocessed Hh is retained in the ER (Figure 3G). Unlike HhN, HhUHA did not accumulate significantly in the Golgi, labeled with the cis-Golgi marker GMAP [24] (Figure 3G). These findings suggest that Hh may require autoproteolysis for efficient exit from the ER. Indeed, it was previously suggested that particular mutations in *sonic hedgehog* (*Shh*), one of the human *hh* homologs, result in defective processing of Shh protein and accumulation of unprocessed Shh in the ER [25]. Taken together, these findings strongly suggest that Hh autoprocessing occurs in the ER.

As these findings suggested that both cleavage and cholesterol modification are required for efficient traffic through the secretory pathway, we then examined whether Hh processing promotes its secretion. In our system, the addition of exogenous cholesterol increased Hh processing, as evidenced by the increased ratio of processed∶unprocessed Hh (Figure 1C). Exogenous cholesterol also increased the secretion of Hh-Ren, but not HhN-Ren or HhC85SN-Ren (Figure 3F), suggesting that cholesterol-mediated processing promotes the secretion of Hh but not forms of Hh that lack the C-terminal domain. Exogenous cholesterol also increased the secretion of HhC85S-Ren, which lacks the palmitoylation site but is still able to undergo autoprocessing (Figure S3). Thus, despite the addition of a lipid-anchor, the Hh processing reaction increases the rate of secretion, possibly by promoting both ER exit and efficient traffic through the Golgi.

### Novel regulators of protein secretion

**Figure 4:**
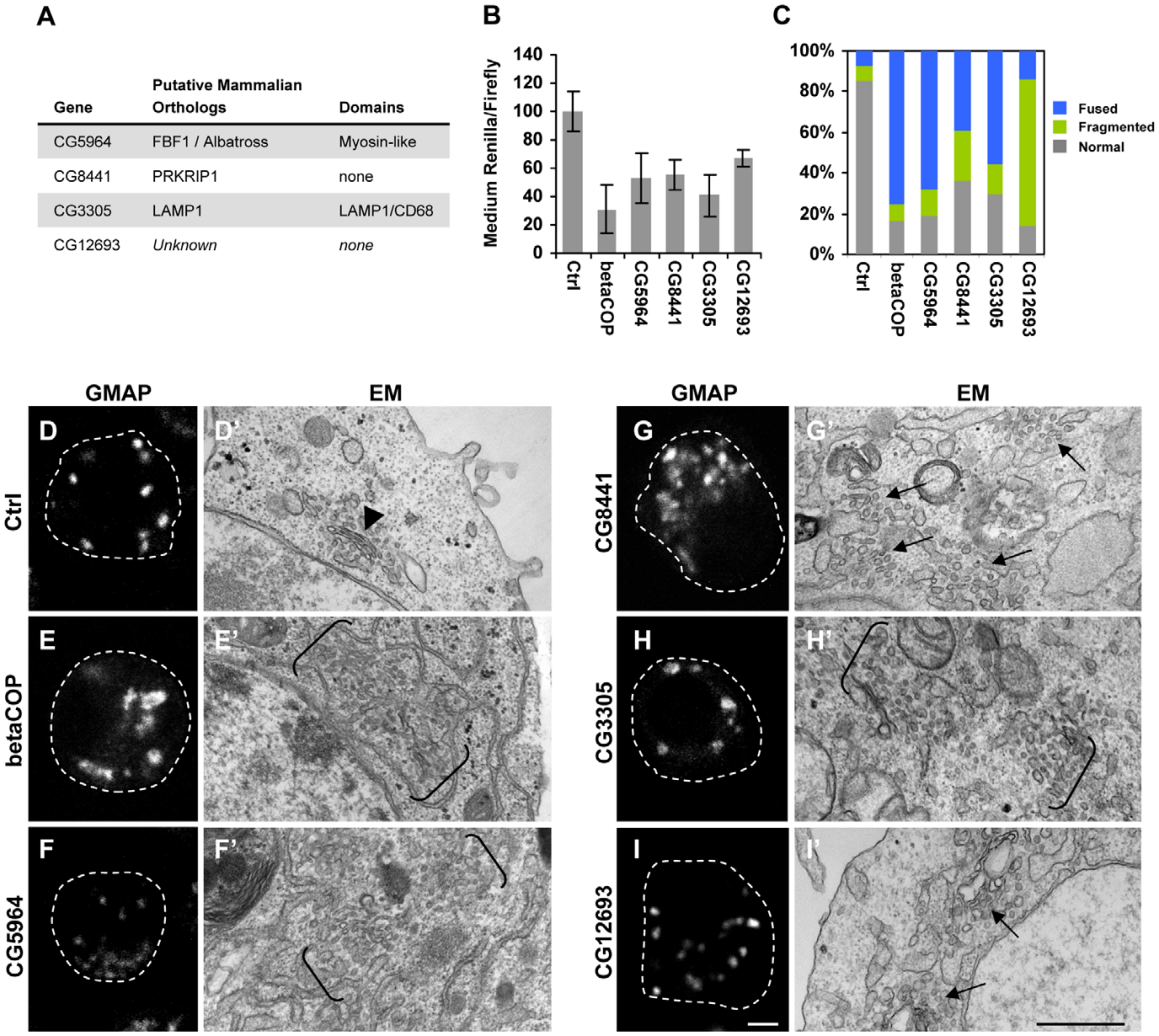
Identification of genes regulating intracellular protein traffic and Golgi structure. (**A**) Putative mammalian orthologs and functional domains of candidate genes considered for further characterization. (**B**) S2 cells transiently transfected with pMT-Ren were cultured for 5 days with the indicated dsRNAs and treated as in Figure 2F. The bars represent the mean medium *Renilla* activity normalized by the lysate firefly activity ± SD. (**C**) S2 cells were transfected with MannII-GFP and cultured with dsRNAs against the indicated genes for 5 days. MannII-GFP staining was quantified by counting the number of cells displaying a normal, fused, or fragmented MannII-GFP pattern (see examples in Figure S5), expressed as a percentage of the total cells counted (100–200 cells per treatment). (**D–I**) S2 cells were cultured with dsRNAs against the indicated genes for 5 days and processed for GMAP immunostaining (red). The control cells in D were treated with Ci dsRNA. Scale bar, 3 µm. (**D′–I′**) S2 cells treated with dsRNA for 5 days were analyzed by EM. Note the intact Golgi stacks in D′ (arrow head) compared to the disrupted Golgi structures (arrows) or elongated fragmented Golgi remnants (between brackets) in other panels. Scale bar, 0.5 µm.

Our screen identified several genes with no previously described role in secretion. We chose to examine four of these novel candidates in more detail (Figure 4A), based on the availability of multiple transgenic RNAi lines at the time for *in vivo* validation (see below). To assess whether these candidate genes regulate general secretion, we examined the effect of depletion of these four candidate genes on the export of a secreted *Renilla* construct in S2 cells. Depletion of all four genes caused a decrease in *Renilla* secretion (Figure 4B), though not the same extent as known components of the general secretion machinery. We also re-screened the other hits from the primary screen with no known role in general secretion using this system and found that almost all were required for *Renilla* secretion (Figure S4), suggesting that these genes may regulate general secretion.

As these findings suggested a more general role for these genes in protein/membrane traffic, we examined the effect of the depletion of the four candidate genes on ER-Golgi trafficking and Golgi structure using immunofluorescence and electron microscopy (EM). Under normal conditions, the transmembrane MannosidaseII-GFP (MannII-GFP) traffics via the ER to the medial-Golgi [18], [26], and localizes immediately adjacent to the cis-Golgi marker GMAP (Figure S5A and S5D) [24]. GMAP is a peripheral Golgi protein that does not traffic via the ER to reach the Golgi. When ER-Golgi traffic is compromised, MannII-GFP is retained in the ER and is dissociated from GMAP, which appears as diffuse staining likely representing GMAP targeted to fragmented Golgi membranes (Figure S5B and S5E). We also observed cells displaying fragmented Golgi, where numerous MannII-GFP/GMAP-positive structures are observed. We quantified the effect of depletion of the 4 candidate genes on MannII-GFP staining by counting the percentage of cells displaying a normal, diffuse, or fragmented MannII-GFP pattern (Figure 4C). Depletion of CG5964 and CG3305 caused predominantly fused MannII-GFP staining, similar to the effect of betaCOP dsRNA, suggesting that ER-Golgi traffic was compromised. Depletion of CG12693, on the other hand, resulted in highly fragmented MannII-GFP distribution. CG8441 depletion resulted in cells displaying either fused or fragmented MannII-GFP staining. Thus, all four candidate genes appear to be required for ER-Golgi traffic and/or maintenance of Golgi morphology.

To further assess the potential role of these four genes on Golgi morphology, we examined the effect of their depletion on GMAP immunofluorescent staining and on Golgi structure by EM. S2 cells typically contain around 20 Golgi per cell [27], and a confocal section typically contains 8–12 distinct GMAP-positive structures (Figure 4D). By EM, Golgi stacks can be observed surrounded by small vesicles (Figure 4D′), as described previously [28]. BetaCOP dsRNA led to enlarged and diffuse GMAP-positive structures, some with ring-like shapes (Figure 4E). EM analysis of these cells demonstrated a disassembly of the stacked Golgi cisternae to form fragmented, vesiculated Golgi and fused tubular membranes throughout the cell (Figure 4E′). Depletion of CG5964, which contains a putative myosin-like domain, caused diffuse GMAP staining and small GMAP-positive structures were visible, possibly representing fragmented Golgi (Figure 4F). EM analysis of these cells demonstrated vesiculated Golgi structures and fused tubular membranes (Figure 4F′). Depletion of CG8441, which shares some homology to protein kinase R inhibitory protein-1 (PRKRIP1), caused clustered regions of enlarged GMAP-positive structures (Figure 4G). Several large areas containing numerous scattered vesicles could be observed by EM (Figure 4G′), representing disrupted Golgi cisternae. Depletion of CG3305, the *Drosophila* ortholog of lysosome associated protein-1 (LAMP1), had little effect on GMAP staining but led to highly vesiculated subcellular regions observable by EM (Figure 4H–H′). Treatment with dsRNA against CG12693, which contains no obvious functional domains, did not affect GMAP staining (Figure 4I). By EM, depletion of CG12693 also showed disrupted Golgi cisterna (Figure 4I′), though to a lesser extent than the other dsRNA treated cells. The cisterna fragments sometimes appeared dilated surrounded by larger vesicles compared to control Golgi vesicles. Together, our findings indicate that these four genes likely have a more general effect on membrane trafficking and/or protein secretion.

### *In vivo* validation using dsRNA transgenics

**Figure 5:**
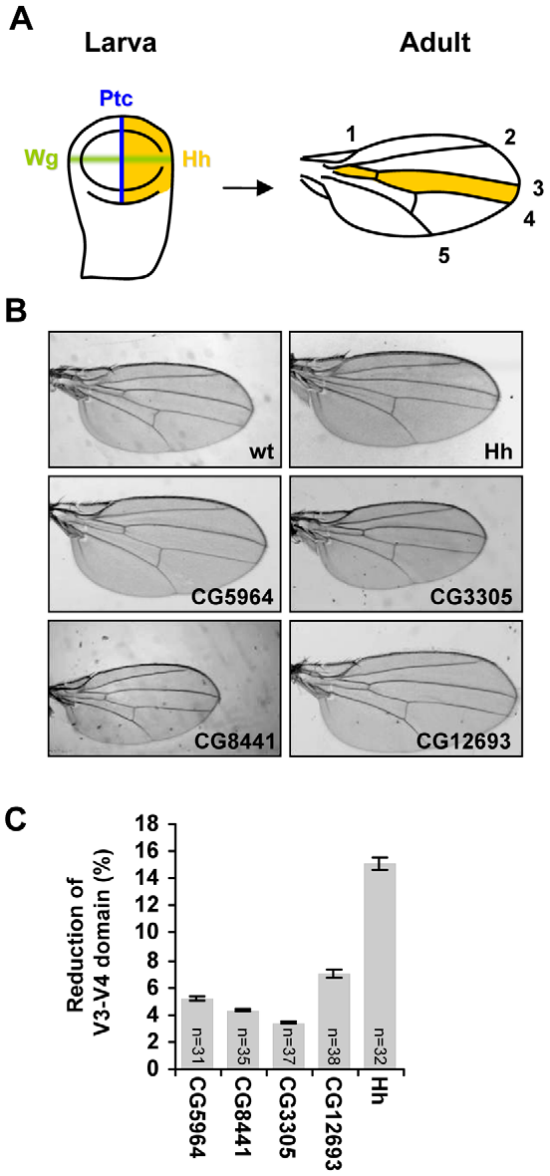
*In vivo* validation of putative regulators of protein secretion using dsRNA transgenics. (**A**) Wg (green) and Hh (yellow) expression domains in the larval imaginal disc and the area patterned by Hh in the adult wing. (**B**) The wing phenotypes of adult flies expressing the indicated *UAS-dsRNA* under the control of *en-Gal4*. (**C**) Quantification of the wing intervein 3–4 domain. The intervein domain area for each wing was measured and normalized over total wing area. Results are shown as the mean percent reduction of the vein 3–4 domain relative to the control ± SD. “n” indicates the number of wings analyzed for each genotype.

To examine the role of these novel regulators of protein secretion *in vivo*, we employed transgenic *Drosophila* lines expressing particular RNA hairpins under the control of the *UAS* sequence, which allows for tissue-specific dsRNA expression. In the wing imaginal disc, the presumptive tissue of the adult wing, Hh is produced in the posterior compartment and patterns, after secretion, the anterior domain of the disc. This domain of the wing imaginal disc corresponds to the region between veins 3 and 4 of the adult wing (Figure 5A). Therefore, the area of the intervein space between veins 3 and 4 provides a readout of Hh activity. To assess the function of our novel candidates, *en-Gal4* or *hh-Gal4* drivers were used to direct the expression of the *UAS-dsRNA* specifically in Hh-producing cells (Figure 5A). The progeny were examined for reduction of the intervein space between veins 3–4 of the adult wing. As expected, the expression of a dsRNA against *hh* in the posterior compartment reduced the width of the Hh-patterned domain (Figure 5B and C). Using the same driver, the expression of dsRNA against our candidate genes caused a modest but significant reduction of this intervein area (Figure 5B and C, Figure S6, and Table S5) and no obvious accumulation of Hh level in posterior wing discs cells (data not shown). We also examined the possibility that overexpression of dsRNA might affect endogenous Hh protein levels by driving dsRNA expression in the dorsal compartment of the wing disc using *apterous-Gal4* driver. Comparing Hh levels in the dorsal and ventral compartments demonstrated no decrease nor accumulation of Hh protein upon dsRNA expression against our candidates (Figure S7).

**Figure 6:**
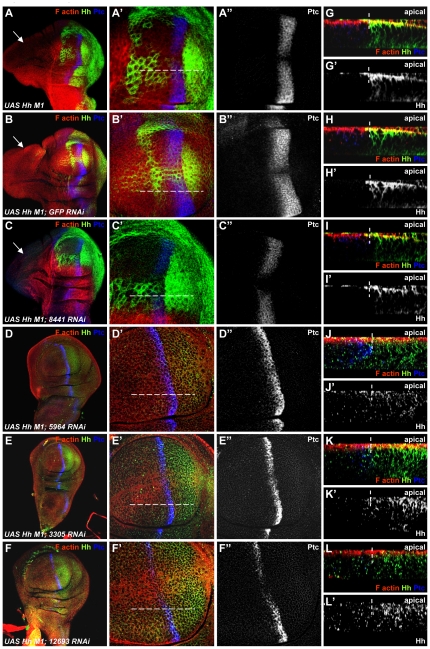
The silencing of CG5964, CG3305, or CG12693 abolishes HhM1-induced anterior outgrowth and alters Hh distribution in producing cells. All panels show confocal images of wing imaginal discs immunostained for Hh (green), Ptc (blue) and phalloidin (red). (**A–F″**) Confocal x/y sections at low (A–F) and high magnifications (A′–F″). Anterior is to the left and dorsal down. The overexpression of Hh from producing cells using *hh-Gal4* generates an anterior outgrowth (arrow in A). Note the broadening of Ptc expression domain (A–A″). The expression of dsRNA against GFP (B–B″), or dsRNA against CG8441 (C–C″) together with HhM1 have no effect on HhM1-induced disc morphology and Hh signalling in receiving cells. In contrast, the overexpression of dsRNAs targeting CG5964, CG3305, or CG12693 prevents HhM1-induced anterior outgrowth (D, E, F). Note that Ptc expression is restored to normal levels in 2 rows of anterior cells (D′–F″ compared to Figure S7A–A′). (**G–I′**) Confocal Z sections showing Hh subcellular distribution when HhM1 is expressed alone (G–G′) or together with dsRNA against GFP (H–H′), or with dsRNA against CG8441 (I–I′). Hh is membranous and localizes mainly to the apical membrane. Note also the high accumulation of Hh at the apical plane of receiving cells. (**J–L′**) The silencing of CG5964 (J–J′), CG3305 (K–K′), and CG12693 (L–L′) alters Hh distribution. Hh is patchy and appears to be distributed over the entire apical/basal axis. Note also that Hh neither accumulates at the apical membrane of producing cells nor at that of receiving cells. Dashed lines mark the position of the compartment boundary (G–L) or position of the Z section within the disc (A′–F′). Apical is up and basolateral is down in panels G–L′.

Several mechanisms have been described that confer robustness to the Hh signalling gradient in the face of altered levels of Hh production [29], [30], [31]. Such homeostatic mechanisms could act to minimize the effect of decreased Hh secretion on wing patterning. Thus, to further validate these candidates *in vivo*, we utilized a Hh-overexpression model, where the overproduction of Hh from posterior cells overrides such homeostatic mechanisms. Hh overexpression in posterior cells led to anterior outgrowth of the wing imaginal disc accompanied by increased target gene expression, such as patched (*ptc*), and adult lethality (Figure 6A–A″). Using this system, we examined the ability of dsRNAs against our candidate genes to rescue the outgrowth and lethality in animals overexpressing Hh. We found that the overexpression of dsRNAs against three of our candidate genes (CG5964, CG3305, and CG12693) in Hh-producing cells prevented the Hh-induced anterior outgrowth (Figure 6D–F). As a consequence, Ptc expression was reduced from 6 to 2 rows of cells in these discs, comparable to the wild type control (Figure 6D′–F″ and Figure S7A–A′). In contrast, dsRNAs against GFP and against CG8441 had no effect on the Hh-induced anterior overgrowth and on the broadening of ptc expression (Figure 6B–C″).

The analysis of Hh distribution in secreting cells showed that Hh is membranous and localizes mainly to the apical membrane (Figure 6G–G′). This subcellular distribution of Hh was not sensitive to the overexpression of dsRNAs against GFP or CG8441. However Hh subcellular localization was altered following the silencing of our three candidate genes (Figure 6J–L′). As a consequence, Hh did not accumulate at the apical membrane of secreting cells, but in contrast, Hh staining presented a punctuate pattern over the entire apical/basal axis. Altogether, these data indicate that our candidate genes are required in secreting cells to regulate Hh activity and Hh subcellular distribution *in vivo*.

**Figure 7:**
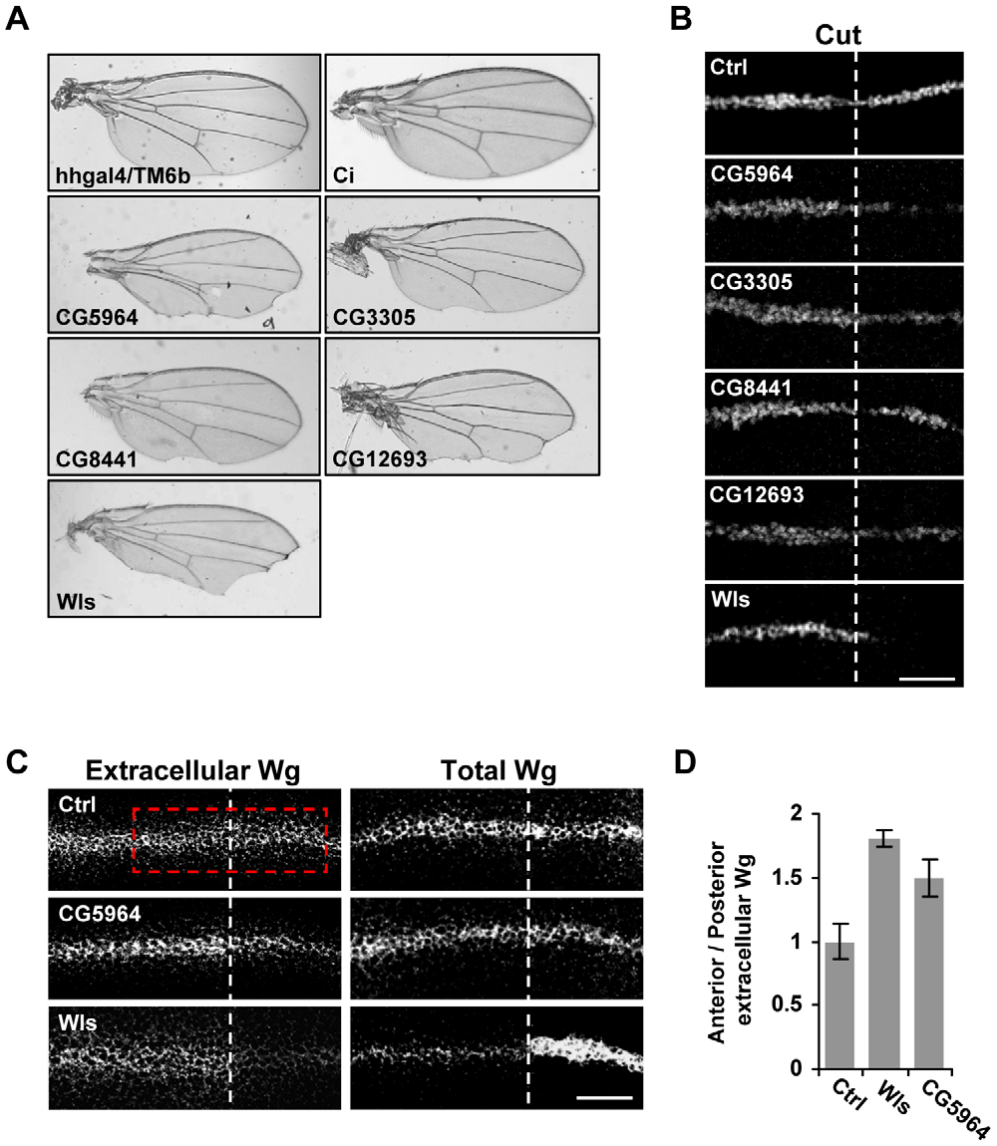
Secretion of Wg is also impaired upon expression of dsRNA against CG5964. (**A**) The wing phenotypes of adult flies expressing the indicated *UAS-dsRNA* under the control of *hh-Gal4*. (**B**) Immunostaining for Cut in wing imaginal discs from flies expressing the indicated *UAS-dsRNA* under the control of *hh-Gal4*. (**C**) Immunostaining for Wg in non-permeabilized (extracellular Wg) and permeabilized (total Wg) wing imaginal discs along the dorso-ventral axis. The broken line indicates the A/P border and the dsRNA was expressed only in the posterior compartment (to the right). The A/P border was determined by anti-Hh staining (not shown). (**D**) Quantification of extracellular Wg staining (see Materials and Methods for details). Bars represent the mean ratio of Wg staining intensity in the anterior divided by the Wg staining intensity of the posterior compartment ± SD for the region marked in red in C. Ctrl: dsRNA against Ci.

During the course of our *in vivo* validation using *UAS-dsRNA* lines using the *hh-Gal4 driver*, we observed wing margin defects reminiscent of a Wg loss of function phenotype. Wg is a lipid-modified morphogen produced in a band of cells along the dorso-ventral (D/V) axis of the *Drosophila* wing imaginal disc (Figure 5A) and is necessary for the expression of the *cut* gene in 2–3 rows of cells along this axis [32]. Using the *hh-Gal4* driver, we therefore analyzed the effect of depletion of our candidate genes on wing morphology and Cut expression. In this way, only the posterior cells are affected by the dsRNA, and the anterior cells serve as an internal control. We found that depletion of our four candidate genes resulted in wing notching along the posterior wing margin (Figure 7A). Similarly, depletion of wntless (wls), which is required for Wg secretion [33], also caused posterior wing margin notching. On the other hand, expression of dsRNA against Ci (which is not expressed in the posterior compartment) did not affect wing morphology. By immunofluorescence, we found that Cut staining was significantly reduced specifically in the posterior cells upon silencing of our 4 candidates (Figure 7B). Similarly, depletion of wls also significantly decreased Cut levels. The observed decrease in Wg signaling was not due to reduced Wg protein levels upon dsRNA overexpression (Figure S8). These findings suggest that Wg signalling is reduced by depletion of our candidate genes.

Since depletion of CG5964 resulted in a severe wing defect and a strong depletion of Cut expression compared to the other candidate genes, we examined whether CG5964 is required for Wg secretion. We analyzed the effect of CG5964 depletion on extracellular and total Wg staining. Expression of dsRNA against CG5964 led to a decrease in extracellular Wg staining with no effect on total Wg levels by conventional staining (Figure 7C and 7D). In our control experiment, expression of dsRNA against Ci did not affect the levels of extracellular Wg in the posterior wing disc. Wls dsRNA also decreased extracellular Wg staining in the posterior compartment, but led to a significant accumulation of Wg by conventional staining, suggesting a different mechanism of action than CG5964. Taken together, these findings indicate a physiological role for CG5964 in the secretion of Wg.

## Discussion

Our findings demonstrate that Hh secretion is largely dependent on the general secretion machinery for its passage through the ER and Golgi. Interestingly, cholesterol-modified Hh secretion is strongly dependent on COPI but not COPII vesicles for its release from the cell. Based on work done mostly in yeast, it has been thought for some time that exit from the ER absolutely required COPII components. However, recent work has suggested that higher organisms might have several mechanisms for protein exit from the ER [34], [35], [36], [37], [38]. As there is evidence that some membrane-anchored proteins can be sorted upon exit from the ER [21], it is plausible that lipid-modification targets Hh to a COPII-independent route out of the ER. Alternatively, we cannot discount the possibility that cholesterol modification targets Hh more efficiently to COPII vesicles, and that a small number of COPII vesicles may still be present following Sec23 dsRNA treatment that are sufficient for release of cholesterol-modified Hh but not other cargo. More detailed analysis of Hh containing structures exiting the ER should shed light on the nature of the ER-to-Golgi transport of Hh protein.

We were unable to confirm the differential roles of COPI versus COPII vesicles in Hh secretion *in vivo* owing to the cell lethality induced by depletion of these vesicle components during development. This lethality was observed under different conditions even when the expression of dsRNA was induced only for a short period of time using the gal80-ts system (data not shown).

Due to the role of COPI in retrograde traffic from the Golgi to ER it is tempting to speculate that Hh requires the recycling of some factor from the Golgi in order for its release from the ER. Along these lines, our screen also identified the KDEL receptor (KdelR), which mediates the retrieval of ER proteins from the Golgi [39]. Indeed, ER to Golgi transport of GPI-linked proteins in yeast is strongly inhibited in mutants of the COPI subunit alphaCOP, suggesting that retrieval of some specific factors from the Golgi to the ER may be required for ER exit of GPI-linked proteins [40]. However, we found that the secretion of our control secreted *Renilla* was equally affected by COPI depletion, indicating that COPI-mediated traffic plays a more general role in anterograde protein traffic in *Drosophila*. Similarly, several COPI components and KdelR were also identified in a screen for regulators of general protein secretion [18], confirming the requirement of COPI-mediated transport for anterograde movement of secreted cargo.

It is currently not known where in producing cells Hh autoproteolysis occurs. Since a hydroxyl-oxygen of cholesterol is responsible for the cleavage of Hh [41], both autoproteolysis and cholesterol modification are intimately linked and occur in the same cellular compartment. The finding that HhN is detected in the ER and Golgi suggests that Hh cholesterol modification occurs before exiting the Golgi and that this modification promotes exit from both the Golgi and ER. In addition, the finding that an uncleavable form of Hh accumulates in the ER suggests that the autoprocessing reaction is required for efficient ER exit, and thus likely take place in the ER. Concordantly, forms of Hh defective for autoprocessing have been suggested to be retained in the ER based on their glycosylation status [25]. It should be noted that HhU is secreted to the plasma membrane [42], indicating that cleavage is not an absolute requirement for ER exit. Indeed, our data do not exclude the possibilities that Hh processing occurs in the Golgi and that unprocessed Hh is recycled back to the ER. However, it is also possible that cleavage and cholesterol modification promote ER exit by a particular route and that in the absence of processing, HhN or HhU eventually exit the ER by COPII-dependent “bulk flow” secretion. Along these lines, the fact that Hh secretion can still occur upon depletion of COPII subunits supports the notion that the autoprocessing reaction might take place in the ER and allows targeting of cholesterol-modified Hh towards an alternative mode of ER exit. Indeed, during the course of our manuscript preparation, a recent study provided convincing biochemical evidence that Hh processing does take place in the ER [43]. Taken together, these findings indicate that Hh autoprocessing occurs in the ER and mediates ER exit.

Our findings suggest that the processing (cleavage and cholesterol-modification) of Hh promotes its movement through the secretory pathway, resulting in increased Hh secretion. This finding is particularly relevant to the understanding how particular mis-sense mutations of *Shh* lead to holoprosencephaly (HPE) [44], [45], [46]. It was previously demonstrated that particular mutations in *Shh* associated with HPE result in defective processing of Shh protein [25]. Recently, a study published during preparation of our manuscript showed rapid degradation of the full-length Hh precursor in the ER [43].. Along with the data presented here, these findings support the conclusion that a decrease in processing would cause a decrease in Shh secretion as a result of the accumulation and degradation of unprocessed Shh in the ER.

How might the addition of a lipid moiety promote secretion of a protein? Hh lipid-modification may target Hh to lipid microdomains (rafts) [8], [47], which could promote recruitment to particular ER exit sites and allow more efficient movement through the Golgi than non-membrane bound cargo. Alternatively, cholesterol modification is necessary for the formation of Hh oligomers [8], [9], [10], [48], which may facilitate the release of Hh. Indeed, the aggregation of certain proteins is necessary for their packaging into secretory granules [49], and aggregation is required for the apical sorting of some proteins [50], [51].

Our screen is the first genome-wide RNAi screen to uncover a differential requirement for COPI and COPII in protein secretion. Previous genome-wide RNAi screens in *Drosophila* cells have uncovered a requirement for COPI and not COPII components in other biological processes: the formation of lipid droplets [52], [53], and viral infection [54]. In the case of viral infection, COPI depletion was proposed to mediate its effect independently of the Golgi as depletion of COPII or Syn5 affected Golgi structure without affecting viral replication. In the case of lipid droplet formation, COPI was proposed to function directly on the surface of lipid droplets as Arf79F, the ADP-ribosylation factor (Arf) small GTPase responsible for COPI vesicle formation, was localized to the surface of lipid droplets. Therefore, unlike Hh secretion, neither lipid droplet formation nor viral infection seems to required ER-to-Golgi transport.

What does our study bring to our understanding of Hh morphogen activity? Morphogen production is inherently linked to morphogen action. Producing cells can regulate morphogen action by controlling the rate of signal secretion, the apico-basal position of signal release, the packaging of the signal, and the activity of the signalling molecule. Therefore, the spatial and temporal regulation of pathway activation is mediated by events occurring in the signal producing cells. The finding that Hh protein may not follow so called “bulk flow” cargo to exit the ER suggests a specific mode of sorting and/or ER exit. Future studies aimed at characterizing these events may shed light on the sorting and trafficking machinery required to move this uniquely modified protein through the cell. As Hh ligand is produced by numerous cancers, characterization of these specific secretion events could uncover specific targets for interfering with Hh secretion.

Our screen identified novel regulators of protein secretion that were not found in two previous screens for regulators of protein secretion in *Drosophila* cells [18], [55]. We demonstrated a physiological role for three of the genes identified in our screen in the developing wing as regulators of Hh and Wg secretion. While many genes identified in these screens await further characterization, the low amount of overlap between them suggest that the experimental systems differed significantly and may offer complementary results in terms of identifying novel genes regulating protein secretion.

## Materials and Methods

### Molecular Biology

*HhN* and wild-type *Hh (HhNp)* cDNAs were subcloned into the *pAct5C* vector (Invitrogen). The QuikChange mutagenesis kit (Stratagene) was used to insert a double restriction site (Hind III and NheI) after the codon 254 in *HhN* and *HhNp* sequences in the *pAct5C* vector. The *Renilla* primers CGG AGG CTT ATG GGCTCC AAA GTG TAC GAC CCC with TGC GCT AGC TTA CGC GCC CTG CTC GTT CTT CAG CAC (for *HhN* construct) or TCG GCT AGC CGC GCC CTG CTC GTT CTT CAG CAC (for *HhNp* construct) were used to amplify PCR the coding region of *Renilla* with a HinDIII site at 5′ and Nhe I site at 3′ PCR fragment. After amplification and digestion with HindIII and NheI, *Renilla* fragments were subcloned into mutated *pAct-HhN* or *pAct-HhNp* to generate *pAct-HhN-Renilla* (HhN-Ren) or *pAct-Hh-Renilla-p* (Hh-Ren) respectively. The C85S mutation was generated by site-specific mutation of *pAct-HhN-Renilla* using the following primer: CCG ATG GTC TTT AGC CCG GCT CAC TCG AGC GGT CCT GGC CGA GGA TTG GGT CGT. The secreted *Renilla* control construct was generated by inserting the *Renilla* coding sequence into the *pMT/Bip/V5/His* vector (Sigma). The secreted *HhSS-Ren* construct used in Figure S1B was generated by fusing the Hh signal sequence to *Renilla*. *HhGFP* and *HhNGFP* were kindly provided by I.Guerrero, *HhUHA* by S.Kunes, *HhHA* by K.Basler, and *MannII-GFP* by V.Malhotra.

### RNAi screen

Using *Drosophila* S2 cells [56], we established a stable cell line expressing the Hh-Ren fusion and a cytoplasmic firefly *luciferase* under the control of the *actin* promoter and used these cells to screen the genome-wide library (DRSC 1.0) at the *Drosophila* RNAi Screening Center (DRSC, Harvard Medical School). All dsRNA sequence information can be found at www.flyrnai.org. Briefly, 1.5×10^4^ cells in serum-free Schneider's medium were plated on white 384-well plates containing 0.25 µg dsRNA for 1 h and incubated for 5 days in complete Schneider's medium supplemented with 1× cholesterol (Gibco) to assure efficient Hh autoprocessing. After 5 days, the medium was replaced and the cells were incubated overnight. The following day, the plates were centrifuged and 20 µl of medium was transferred to new 384-well plates and the *Renilla* activity was measured using a commercially available kit (Promega) on an Analyst GT plate reader (Molecular Devices). The remaining medium was aspirated and the firefly and *Renilla* activity of the cell lysates was also measured. The entire screen was performed in duplicate.

In order to correct for local aberrations and for edge effects, the readings for individual wells were normalized by both their respective row and column averages, followed by log transformation. To identify candidate genes that regulate secretion and release of cholesterol-modified Hh, we examined the effect of each dsRNA on following ratios: A) *Renilla* activity in the medium normalized by firefly activity in the lysate, B) *Renilla* activity in the lysate normalized by firefly activity in the lysate, or C) *Renilla* activity in the medium divided by *Renilla* activity in the lysate. Values for each ratio were transformed to z-scores, and we selected for dsRNAs that caused an increase or decrease of greater than 2 SD from the plate average for both of the two replicate experiments in at least one of the above complementary categories. We eliminated dsRNAs that caused a decrease or increase in intracellular firefly of greater than 2 SD. We eliminated amplicons with 19 bp OT, since in this case the effect of the particular dsRNA could be due to suppression of genes other than the intended target gene [57]. We did select a handful of genes that contained 1–2 19 bp off-targets for further screening based on their possible role in protein secretion. We also eliminated dsRNAs targeting in silico predicted genes (Heidelberg or Sanger annotations), given that the percentage of these sequences that encode proteins is not known. Other genes were filtered out based on their known functions (see Table S2), resulting in the selection of 125 genes for secondary screening (Table S1). One gene, Bap55, was originally considered as a hit but was removed during the course of secondary screening as clear evidence indicates that Bap55 localizes to chromatin remodeling complexes in the nucleus [58], [59].

For secondary screening, PCR templates for candidate genes were obtained from the DRSC that did not overlap with those used in the primary screen. Where possible, multiple amplicons were used targeting the same gene for all subsequent analysis. In some cases, new templates were generated using the corresponding DRSC primer sequences. All sequence and primer information can be found at www.flyrnai.org. PCR templates were transcribed into dsRNA using the T7 MEGAscript kit (Ambion). For secondary screening, wild-type S2 cells were batch transfected overnight in 6-well plates with pAct-firefly and an inducible pMT-Hh-Ren construct. The following day, cells were resuspended in serum free medium and plated on white 96-well plates containing 250 ng dsRNA for 1 h followed by 5 days in complete cholesterol supplemented Schneider's. After 5 days, the medium was replaced with medium containing 1× cholesterol and 100 µM CuSO_4_ and incubated for 36 h, at which point the *luciferase* activities were measured on a Berthold CENTRO LB960 plate reader.

For secondary screen data (Figure 2F), normalized secretion values (n = 5 replicates per plate) are represented as a percent of the controls (n = 5–10 per plate), and *p* values were calculated using the Student's *t*-test. For the data presented in Figures 2, 3, 4, at least 3 independent experiments were performed with n = 5 replicates per experiment. In all cases, samples and controls were randomly distributed on the interior wells of 96-well plates, with the edge wells containing only culture medium. For Figure 3, in order to compare the effects of various conditions on the secretion of different constructs, and thus different transfections, we found it more reliable to use the ratio of extracellular/intracellular *Renilla* as readout for secretion as this controlled for different levels of expression of the different constructs and allowed for the contribution of accumulated proteins within the cell.

### Fluorescent Microscopy

S2 or S2R+ cells were resuspendend in serum-free medium and plated in 8-well chamber slides (Nunc) containing 500 ng dsRNA. After one hour, cells were transfected with the indicated plasmids using Effectene (Qiagen) in complete medium. After 2–5 days, cells were fixed in 4% paraformaldehyde for 10 min, washed in PBS+0.1% Triton (PBT), and incubated for 2–3 h with primary antibodies in PBT+1%BSA. The primary antibodies used were rabbit “Calvados” polyclonal anti-Hh at 1∶400 [10], mouse anti-Golgi (Calbiochem) at 1∶200, anti-GMAP [24] at 1∶250, guinea pig anti-Golgi (gift from S.Munro) at 1∶300, and anti-HA 12CA5 (Boehringer Mannheim) at 1∶100. Samples were subsequently washed with PBT, incubated with FITC, Cy3, and/or Cy5 conjugated secondary antibodies (Jackson Laboratory) diluted 1∶250 in PBT+1%BSA for 1–2 h, washed, and mounted for imaging. Images were acquired using a Leica Sp DMR TCS_NT confocal microscope and analyzed using ImageJ. In all cases, 50–100 transfected cells were analyzed per condition and images shown are representative of at least 50% of cells examined.

Wing imaginal disc immunofluorescence was performed as described previously [9]. Antibodies were used at the following dilutions: mouse 4D4 monoclonal anti-Wg at 1∶50 (Development Studies Hybridoma Bank, University of Iowa; DSHB); mouse 2B10 monoclonal anti-Cut at 1∶200 (DHSB); mouse 5E10 monoclonal anti-Ptc at 1∶400 (DHSB); rabbit “Calvados” polyclonal anti-Hh at 1∶400; rat 2A1 monoclonal anti-Ci at 1∶20 (DSHB), mouse N2 7A1 monoclonal anti-Arm 1∶50 (DSHB). F-actin was stained using phalloidin-tetramethylrhodamine B isothio-cyanate (Sigma). Fluorescent secondary antibodies were used at 1∶100 for Cy5-conjugated goat anti-mouse (Jackson Laboratory), 1∶100 for Cy3-conjugated donkey anti-rat (Molecular Probes), and 1∶500 for Alexa 488-conjugated goat anti-mouse (Molecular Probes). Extracellular labeling was performed essentially as described previously [60], but live discs were dissected and incubated in Schneider's S2 medium with 10% serum. Incubation of live discs with anti-Wg (1∶20) was for 90 min at 4°C. Fluorescent images were obtained with a Leica Sp DMR TCS_NT confocal microscope and processed using Adobe Photoshop 7.0. Images in Figure 7 are optical slices of 1.5–2 µm. To precisely quantify the relative extracellular Wg signal, we developed an algorithm that calculated the integrated Wg signal across a defined width along the dorsal/ventral axis of the wing disc. Using the raw images, the cumulative mean intensity of both Wg and Hh staining was measured along the curvilinear axis. The ratio of anterior/posterior curves for Wg was then extracted, using the Hh signal to precisely define the anterior/posterior boundary. To ensure the robustness of the measure, the system provided controls based on the stability of the Wg anterior/posterior ratio over a large range of distance from anterior/posterior border and on the flatness of the Hh signal to prevent artifacts during acquisition. The system stored the data for a series of measures and exported the mean and SD to a spreadsheet.

### Transmission electron microscopy

For ultrastructural analysis of the Golgi apparatus, the cells treated with dsRNA and controls were fixed in 1.6% glutaraldehyde in 0.1M phosphate buffer, pH 7.5 then washed with 0.1M cacodylate buffer (pH 7.5) and postfixed with 1% osmium tetroxide in cacodylate buffer containing 0.8% potassium ferrocyanide. After dehydration in acetone, samples were embedded in epoxy resin. Ultrathin sections were contrasted conventionally and observed with a Philips CM12 electron microscope fitted with a CCD camera (Morada, Olympus SIS). At least two independent experiments were examined for each condition.

### Western Blotting

Samples were resolved by SDS-PAGE and transferred to nitrocellulose membranes according to standard protocols. Membranes were probed with anti-*Renilla* (Chemicon), anti-*Renilla* (Millipore, gift from S.Laporte), anti-Hh [10], or anti-Fu [61] and visualized on a Fuji LAS 3000.

### *Drosophila* Genetics

The *UAS-dsRNA* lines used were obtained from the Vienna *Drosophila* Screening Center (http://www.vdrc.at/) and from the National Institute of Genetics (R. Ueda, Japan). The various *UAS-dsRNA* lines were crossed with *en-Gal4*, *hh-Gal4*, or *UAS-Dicer2;hh-Gal4* lines to specifically target RNAi expression in the posterior cells of wing imaginal discs, or with *apterous-Gal4* to target RNAi expression in the dorsal cells of wing imaginal discs. The *UAS-hhM1* line was obtained from P. Ingham [62]. Flies were raised and crossed at 25°C according to standard procedures. Wings were dissected, dehydrated in ethanol, and mounted in euparal. Photographs of the wings were digitized with a Zeiss Axioplan 2 microscope, processed and analyzed using Adobe Photoshop software.

## Supporting Information

Figure S1 **Effect of Sec23 and Sar1 depletion on secretion.** (**A**) Schematic representation of the secreted *Renilla* construct containing the Hh signal sequence (HhSS-Ren). (**B**) Sec23 depletion blocks secretion of a secreted *Renilla*. S2 cells transiently transfected with PMT-HhSS-Ren were cultured for 5 days with the indicated dsRNAs and treated as in Figure 2F. The bars represent the mean medium/lysate *Renilla* activity ± SD. Note the strong reduction in *Renilla* secretion upon Sec23 or Sec24 depletion, confirming the ability of these dsRNAs to block general secretion. Depletion of Sec13 or Sec31 had no effect on secretion in our system. (**C**) HhN-Ren is more sensitive than Hh-Ren to Sar1 depletion. S2 cells transiently transfected with PMT-Hh-Ren or PMT-HhN-Ren were cultured for 5 days with the indicated dsRNAs and treated as in Figure 2F. The bars represent the mean medium/lysate *Renilla* activity ± SD.
(TIF)

Figure S2 **HhNGFP and HhN-Ren accumulate in the trans-Golgi.** (**A**) S2R+ cells were transfected with HhGFP or HhNGFP, fixed, and immunostained with anti-GMAP (red). HhNGFP localized to Golgi structures, marked with the cis-Golgi marker GMAP (compare to Figure 4C). (**B**) S2R+ cells were transfected with Hh-Ren or HhN-Ren, fixed, and immunostained with anti-Hh (green) and anti-Golgi (red). HhN-Ren strongly localized to the Golgi and to the perinuclear ER (arrow). Compare with Figure 3E.
(TIF)

Figure S3 **Cholesterol promotes HhC85S-Ren secretion.** (**A**) Schematic representation of the secreted *HhC85S-Renilla* construct, which lacks the palmitoylation site but is still able to undergo autoprocessing. (B) S2R+ cells transiently transfected with an inducible pMT-HhSS-Ren, pMT-Hh-Ren, or pMT-HhC85S-Ren construct were cultured with or without cholesterol concentrate and treated as in Figure 2F. The bars represent the mean medium/lysate *Renilla* activity ± SD.
(TIF)

Figure S4 **Identification of putative regulators of general secretion.** S2 cells transiently transfected with pMT-Ren were cultured for 5 days with the indicated dsRNAs and treated as in Figure 2F. The bars represent the mean medium *Renilla* activity normalized by the lysate firefly activity ± SD. Only CG14804 did not significantly reduce *Renilla* secretion at p<0.05 (NS = not significant).
(TIF)

Figure S5 **MannII-GFP phenotypes.** Examples of normal (A, D), fused (B,E) and fragmented (C,F) MannII-GFP staining. (**A–C**) S2 cells were transfected with MannII-GFP were fixed and stained with phalloidin (red). Normal cells (A) typically display 8–12 MannII-GFP structures per confocal section of a cell, while cell displaying fragmented staining (C) have >20 MannII-GFP positive structures. (**D–F**) S2 cells transfected with MannII-GFP were fixed, and stained with anti-GMAP (red). Normal cells (D) show medial-Golgi MannII-GFP staining adjacent and slightly overlapping with the cis-Golgi GMAP staining (see inset). In cells displaying fused MannII-GFP staining (E), MannII-GFP is retained in the ER and is dissociated from GMAP, which appears as diffuse staining likely representing GMAP targeted to fragmented Golgi membranes. In cells displaying fragmented MannII-GFP (F), the segregation of MannII-GFP and GMAP is lost.
(TIF)

Figure S6 **Effects of silencing candidate genes on Hh pathway activity** ***in vivo*** **.** Quantification of the wing intervein 3–4 domain of adult flies expressing the indicated *UAS-dsRNA* under the control of *en-Gal4* driver. Bars represent the mean V3–V4 intervein domain area as a percentage of the total wing area. The overexpression of the dsRNAs results in a modest but statistically significant reduction of V3–V4 domain. Asterisks denote the significant reduction of the V3–V4 domain (* p<0.005; ** p<0.0003). Error bars represent SEM. P values were calculated with Student's t test. “n” indicates the number of wings analyzed for each genotype.
(TIF)

Figure S7 **Hh levels are not altered by the silencing of candidate genes by the selected dsRNAs.** (A) Analysis of Hh protein levels upon the indicated *UAS-dsRNA* overexpression in the dorsal compartment under the control of *apterous-Gal4* driver. (A–A′) A wild-type disc stained for Hh (green) and Ptc (blue). (B–B′) An *apterous-Gal4/UAS-Hh RNAi* wing disc stained for Hh (green) and Armadillo (Arm, blue). (C–F′) The wing discs show Hh (green), Arm (blue), and Ci (red) immunostaining. In all panels Hh is also shown in white. The arrow (B–B′) points the dramatic reduction of Hh levels upon overexpression of Hh RNAi. The broken line indicates the ventral/dorsal border. (G) Quantification of total Hh levels. Posterior Hh levels were calculated by taking a measurement of signal intensity in ventral and in dorsal compartments and subtracting background levels for each respective compartment (far in anterior). An example of the areas used in a sample disc is shown in A′. No significant difference was observed on Hh levels when dsRNAs against the different candidates were expressed in the dorsal compartment. Bars represent the mean ratio of Hh staining intensity in the ventral compartment divided by Hh staining intensity in the dorsal compartment. Error bars represent SEM. “n” indicates the number of wing discs analyzed for each genotype.
(TIF)

Figure S8 **The novel regulators do not affect Wg protein levels.** Analysis of Wg protein levels along the dorso-ventral axis. (A) Immunostaining for Wg (green) in wing imaginal discs from flies expressing the indicated dsRNAs under the control of *hh-Gal4*. The A/P border was determined by anti-Hh staining (red). (**B**) Quantification of total protein Wg levels (see materials and methods). Bars represent the mean ratio of Wg staining intensity in the anterior divided by the Wg staining intensity of the posterior compartment. No significant difference was observed on Wg levels when dsRNAs against the various candidates were expressed only in the posterior compartment. Error bars represent SEM. “n” indicates the number of wing discs analyzed for each genotype. Controls: dsRNA against Ci and against Wls.
(TIF)

Table S1 Hits selected for secondary screening.
(XLS)

Table S2 Hits from primary screen eliminated from further screening.
(XLS)

Table S3 Hits eliminated from secondary screening based on effect on cytoplasmic firefly activity.
(XLS)

Table S4 COP amplicons used in secondary screening.
(XLS)

Table S5 List of the different transgenic RNAi lines used in this study.
(XLS)
