# Supplementary figures and images for: A Genome-Wide RNAi Screen Identifies Regulators of Cholesterol-Modified Hedgehog Secretion in Drosophila
Source: PLoS One. 2012 Mar 14;7(3):e33665. doi: 10.1371/journal.pone.0033665 (PMC3303847; doi:10.1371/journal.pone.0033665)

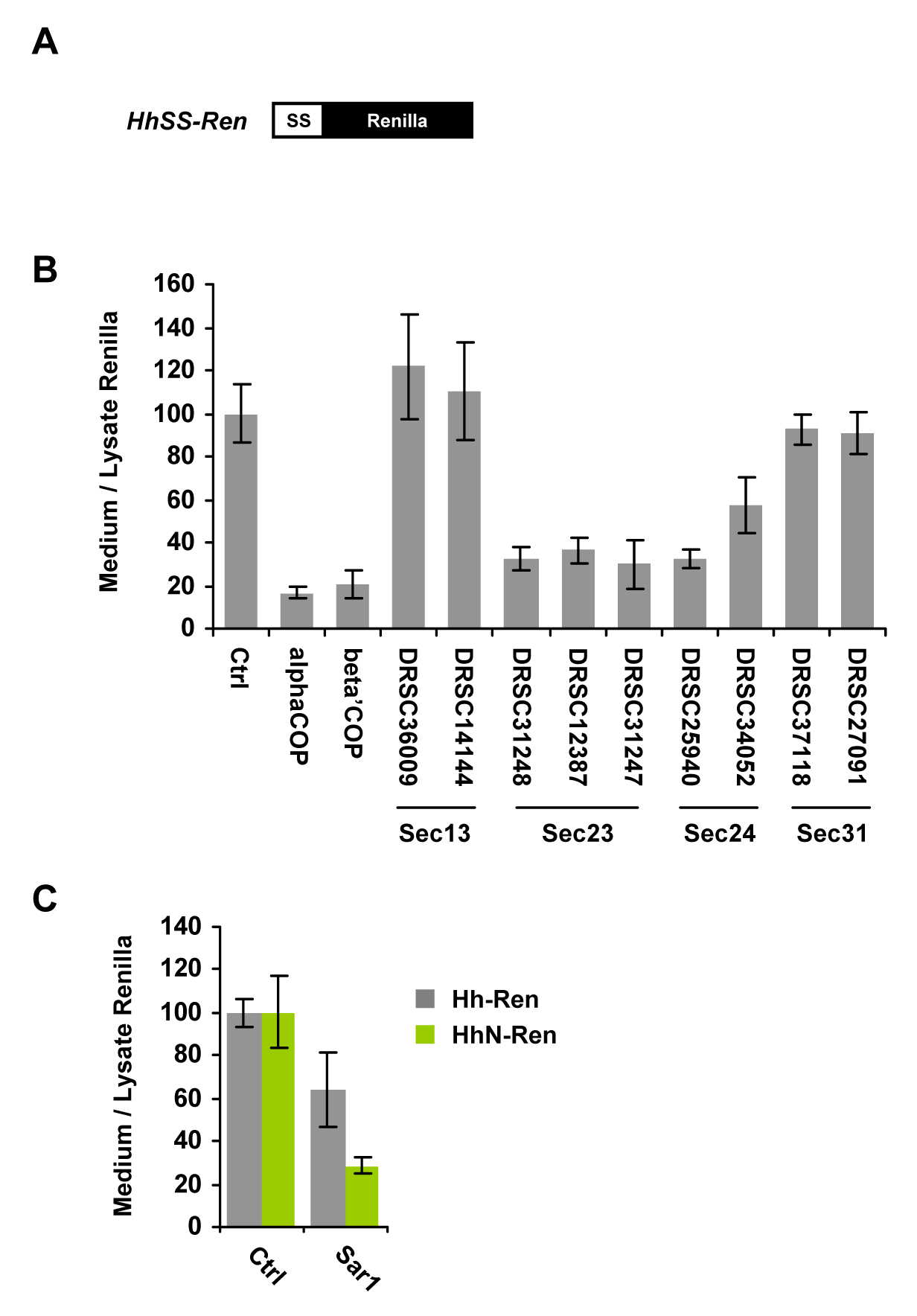

Supplement: Figure S1 — Effect of Sec23 and Sar1 depletion on secretion. (A) Schematic representation of the secreted Renilla construct containing the Hh signal sequence (HhSS-Ren). (B) Sec23 depletion blocks secretion of a secreted Renilla. S2 cells transiently transfected with PMT-HhSS-Ren were cultured for 5 days with the indicated dsRNAs and treated as in Figure 2F. The bars represent the mean medium/lysate Renilla activity ± SD. Note the strong reduction in Renilla secretion upon Sec23 or Sec24 depletion, confirming the ability of these dsRNAs to block general secretion. Depletion of Sec13 or Sec31 had no effect on secretion in our system. (C) HhN-Ren is more sensitive than Hh-Ren to Sar1 depletion. S2 cells transiently transfected with PMT-Hh-Ren or PMT-HhN-Ren were cultured for 5 days with the indicated dsRNAs and treated as in Figure 2F. The bars represent the mean medium/lysate Renilla activity ± SD. (TIF) [file pone.0033665.s001.tif]

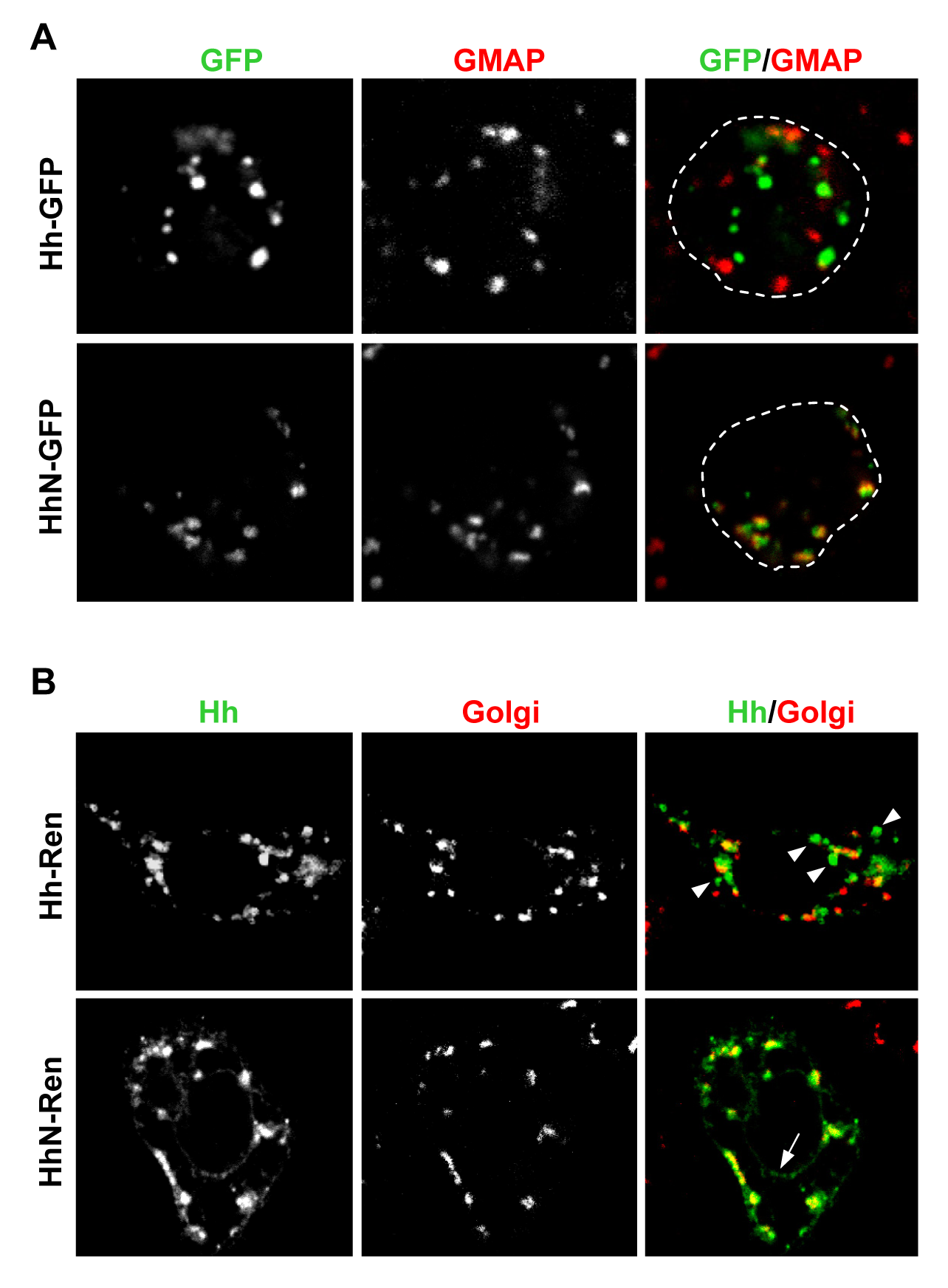

Supplement: Figure S2 — HhNGFP and HhN-Ren accumulate in the trans-Golgi. (A) S2R+ cells were transfected with HhGFP or HhNGFP, fixed, and immunostained with anti-GMAP (red). HhNGFP localized to Golgi structures, marked with the cis-Golgi marker GMAP (compare to Figure 4C). (B) S2R+ cells were transfected with Hh-Ren or HhN-Ren, fixed, and immunostained with anti-Hh (green) and anti-Golgi (red). HhN-Ren strongly localized to the Golgi and to the perinuclear ER (arrow). Compare with Figure 3E. (TIF) [file pone.0033665.s002.tif]

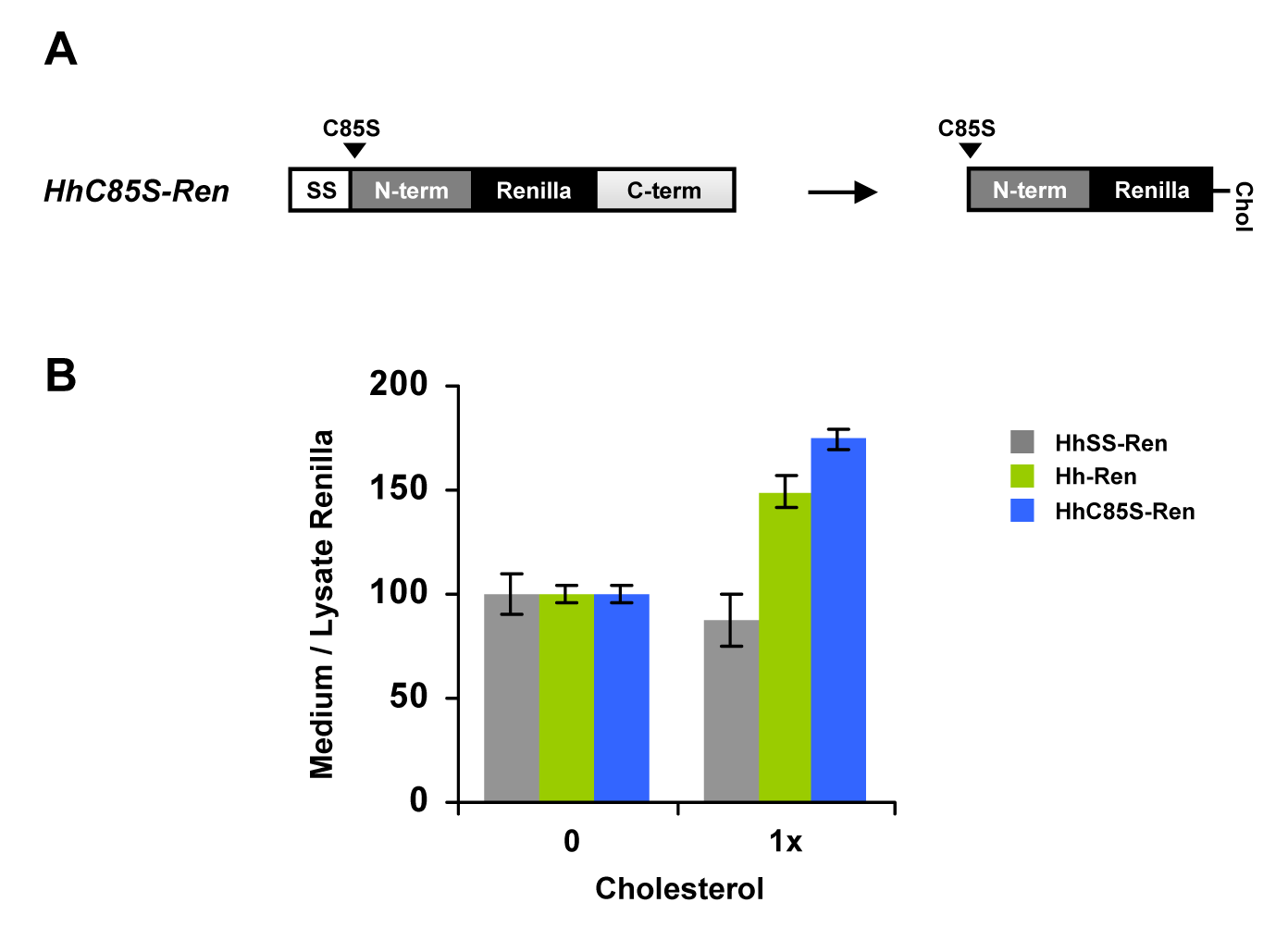

Supplement: Figure S3 — Cholesterol promotes HhC85S-Ren secretion. (A) Schematic representation of the secreted HhC85S-Renilla construct, which lacks the palmitoylation site but is still able to undergo autoprocessing. (B) S2R+ cells transiently transfected with an inducible pMT-HhSS-Ren, pMT-Hh-Ren, or pMT-HhC85S-Ren construct were cultured with or without cholesterol concentrate and treated as in Figure 2F. The bars represent the mean medium/lysate Renilla activity ± SD. (TIF) [file pone.0033665.s003.tif]

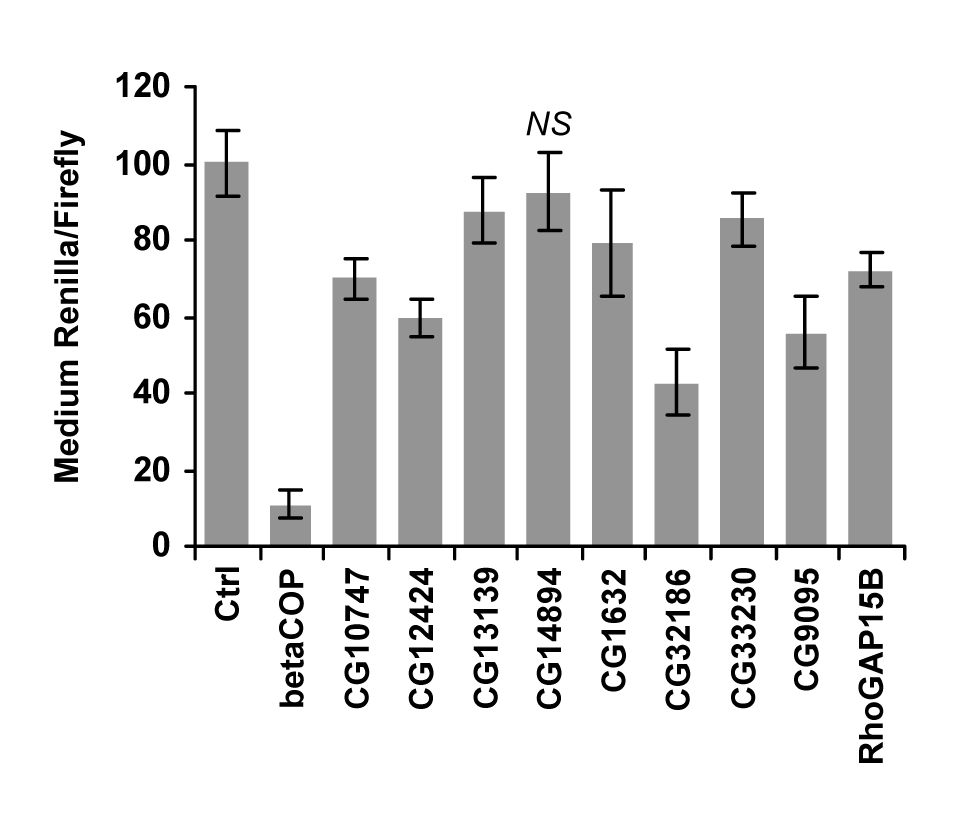

Supplement: Figure S4 — Identification of putative regulators of general secretion. S2 cells transiently transfected with pMT-Ren were cultured for 5 days with the indicated dsRNAs and treated as in Figure 2F. The bars represent the mean medium Renilla activity normalized by the lysate firefly activity ± SD. Only CG14804 did not significantly reduce Renilla secretion at p<0.05 (NS = not significant). (TIF) [file pone.0033665.s004.tif]

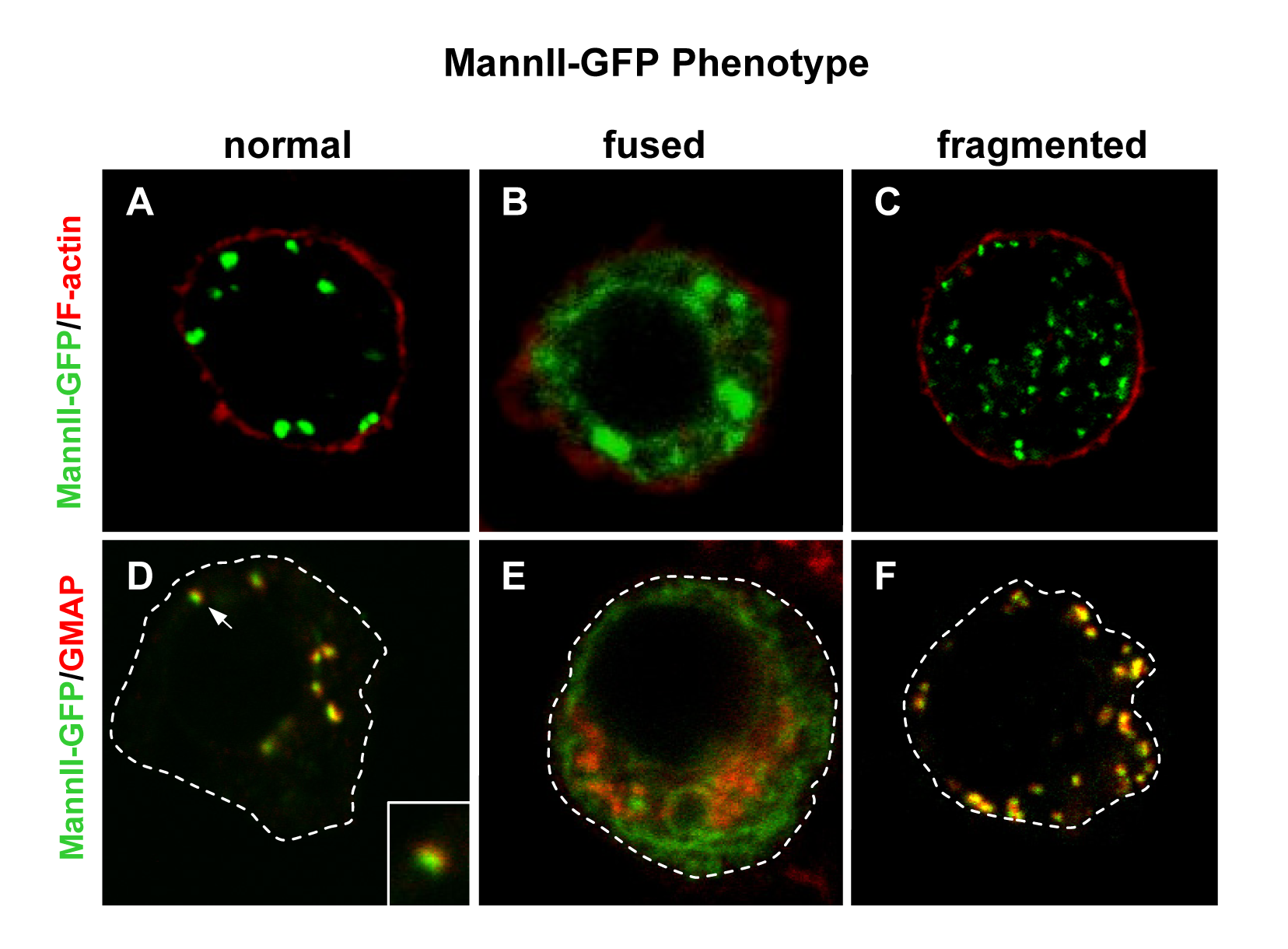

Supplement: Figure S5 — MannII-GFP phenotypes. Examples of normal (A, D), fused (B,E) and fragmented (C,F) MannII-GFP staining. (A–C) S2 cells were transfected with MannII-GFP were fixed and stained with phalloidin (red). Normal cells (A) typically display 8–12 MannII-GFP structures per confocal section of a cell, while cell displaying fragmented staining (C) have >20 MannII-GFP positive structures. (D–F) S2 cells transfected with MannII-GFP were fixed, and stained with anti-GMAP (red). Normal cells (D) show medial-Golgi MannII-GFP staining adjacent and slightly overlapping with the cis-Golgi GMAP staining (see inset). In cells displaying fused MannII-GFP staining (E), MannII-GFP is retained in the ER and is dissociated from GMAP, which appears as diffuse staining likely representing GMAP targeted to fragmented Golgi membranes. In cells displaying fragmented MannII-GFP (F), the segregation of MannII-GFP and GMAP is lost. (TIF) [file pone.0033665.s005.tif]

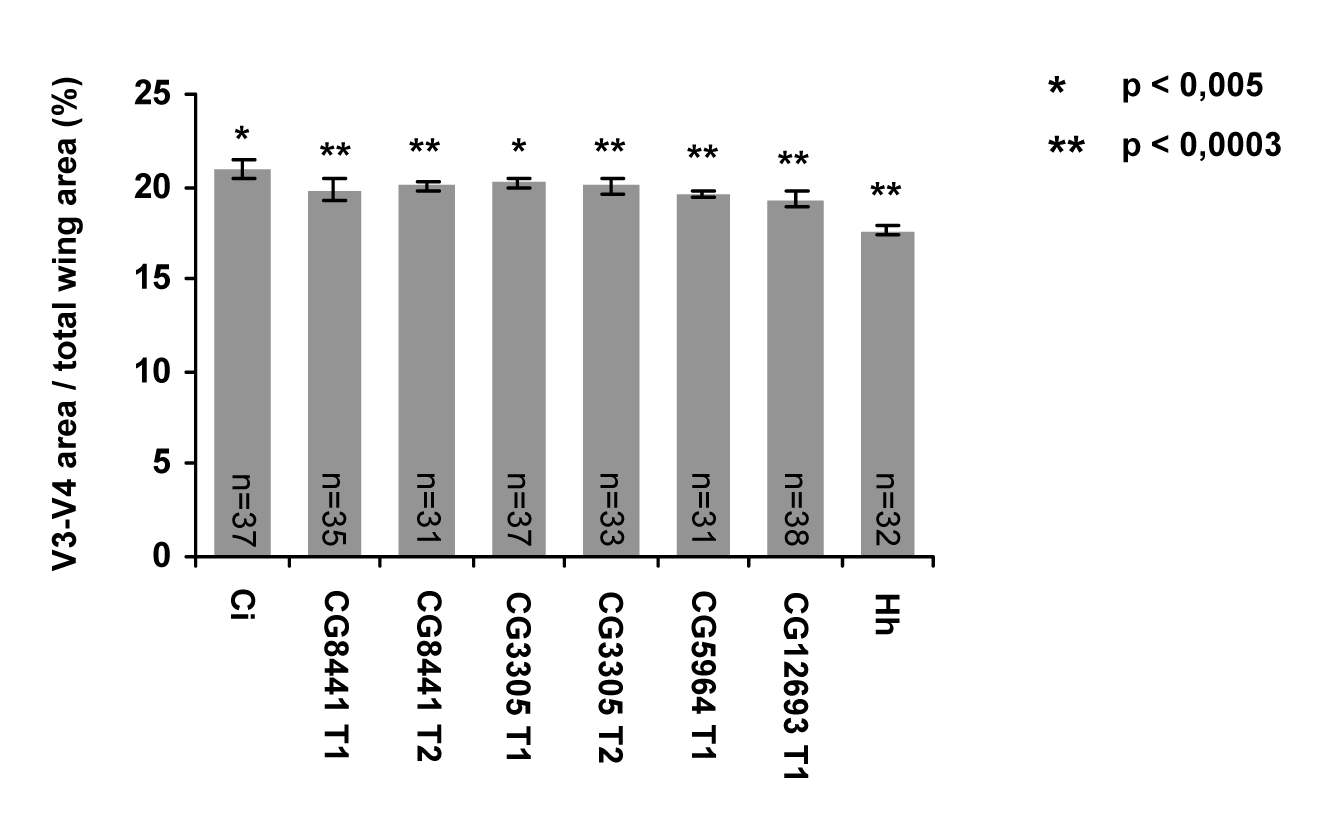

Supplement: Figure S6 — Effects of silencing candidate genes on Hh pathway activity in vivo . Quantification of the wing intervein 3–4 domain of adult flies expressing the indicated UAS-dsRNA under the control of en-Gal4 driver. Bars represent the mean V3–V4 intervein domain area as a percentage of the total wing area. The overexpression of the dsRNAs results in a modest but statistically significant reduction of V3–V4 domain. Asterisks denote the significant reduction of the V3–V4 domain (* p<0.005; ** p<0.0003). Error bars represent SEM. P values were calculated with Student's t test. “n” indicates the number of wings analyzed for each genotype. (TIF) [file pone.0033665.s006.tif]

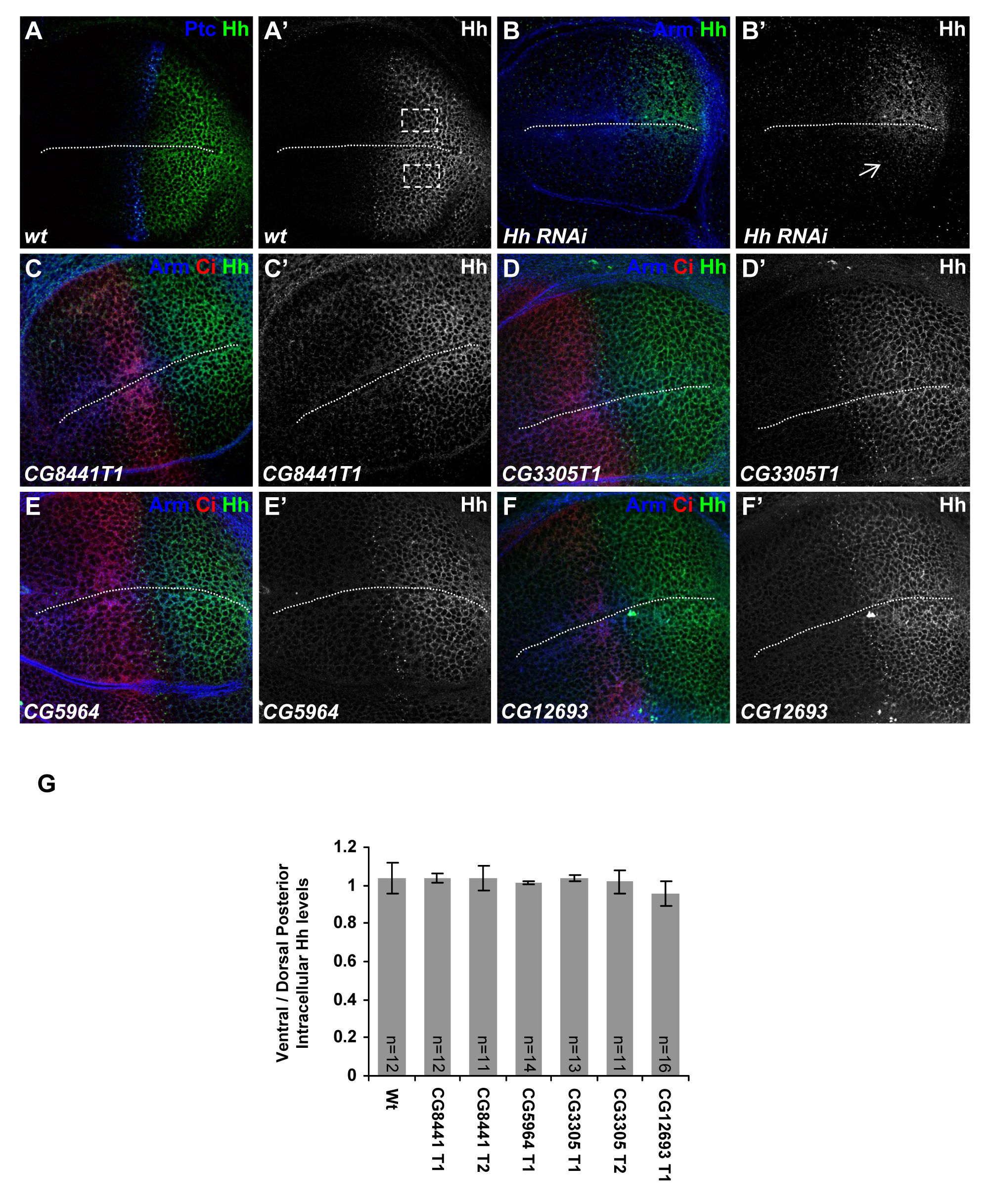

Supplement: Figure S7 — Hh levels are not altered by the silencing of candidate genes by the selected dsRNAs. (A) Analysis of Hh protein levels upon the indicated UAS-dsRNA overexpression in the dorsal compartment under the control of apterous-Gal4 driver. (A–A′) A wild-type disc stained for Hh (green) and Ptc (blue). (B–B′) An apterous-Gal4/UAS-Hh RNAi wing disc stained for Hh (green) and Armadillo (Arm, blue). (C–F′) The wing discs show Hh (green), Arm (blue), and Ci (red) immunostaining. In all panels Hh is also shown in white. The arrow (B–B′) points the dramatic reduction of Hh levels upon overexpression of Hh RNAi. The broken line indicates the ventral/dorsal border. (G) Quantification of total Hh levels. Posterior Hh levels were calculated by taking a measurement of signal intensity in ventral and in dorsal compartments and subtracting background levels for each respective compartment (far in anterior). An example of the areas used in a sample disc is shown in A′. No significant difference was observed on Hh levels when dsRNAs against the different candidates were expressed in the dorsal compartment. Bars represent the mean ratio of Hh staining intensity in the ventral compartment divided by Hh staining intensity in the dorsal compartment. Error bars represent SEM. “n” indicates the number of wing discs analyzed for each genotype. (TIF) [file pone.0033665.s007.tif]

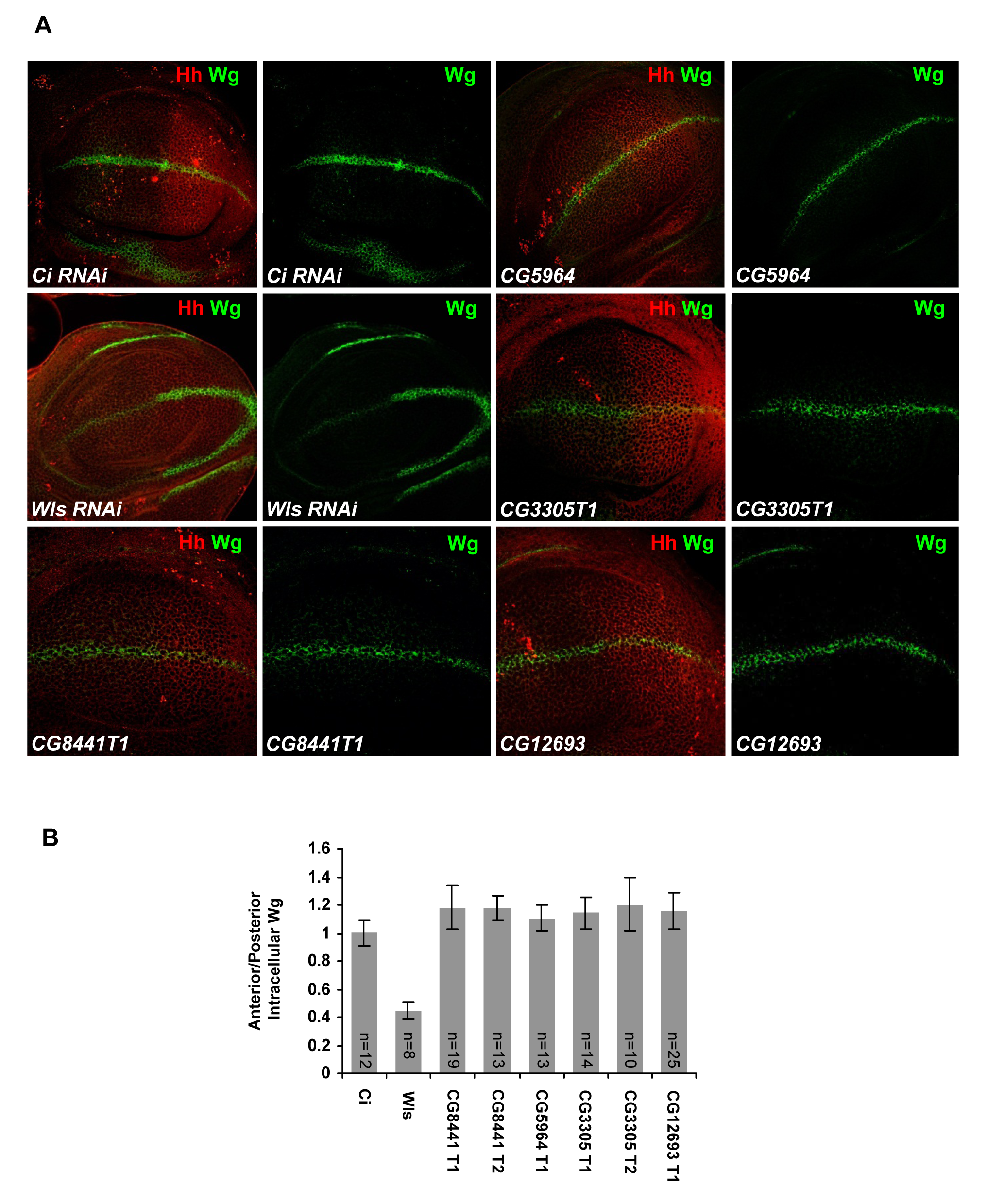

Supplement: Figure S8 — The novel regulators do not affect Wg protein levels. Analysis of Wg protein levels along the dorso-ventral axis. (A) Immunostaining for Wg (green) in wing imaginal discs from flies expressing the indicated dsRNAs under the control of hh-Gal4. The A/P border was determined by anti-Hh staining (red). (B) Quantification of total protein Wg levels (see materials and methods). Bars represent the mean ratio of Wg staining intensity in the anterior divided by the Wg staining intensity of the posterior compartment. No significant difference was observed on Wg levels when dsRNAs against the various candidates were expressed only in the posterior compartment. Error bars represent SEM. “n” indicates the number of wing discs analyzed for each genotype. Controls: dsRNA against Ci and against Wls. (TIF) [file pone.0033665.s008.tif]
